# Affective dysregulation in childhood - optimizing prevention and treatment: protocol of three randomized controlled trials in the ADOPT study

**DOI:** 10.1186/s12888-019-2239-8

**Published:** 2019-09-02

**Authors:** Manfred Döpfner, Josepha Katzmann, Charlotte Hanisch, Jörg M. Fegert, Michael Kölch, Anne Ritschel, Anne-Katrin Treier, Martin Hellmich, Veit Roessner, Ulrike Ravens-Sieberer, Tobias Banaschewski, Anja Görtz-Dorten, on behalf of the ADOPT Consortium, Pascal Aggensteiner, Dorothee Bernheim, Stefanie Bienioschek, Daniel Brandeis, Maurice Breier, Veronika Dobler, Franziska Frenk, Franziska Giller, Claudia Ginsberg, Monja Groh, Stefan Heintz, Sarah Hohmann, Christine Igel, Michaela Junghänel, Anna Kaiser, Betül Katmer-Amet, Katrin Koppisch, Kristin Kuhnke, Sabina Millenet, Kristina Mücke, Theresa Nickel, Christiane Otto, Elisaveta Rodova-Ghasemi, Angelina Samaras, Anne Schreiner, Jennifer Schroth, Anne Schüller, Marie Steiner, Marion Steiner, Susanne Steinhauser, Matthias Winkler, Anne Wüstner, Sara Zaplana

**Affiliations:** 1 Department of Child and Adolescent Psychiatry, Psychosomatics and Psychotherapy, Medical Faculty of the University of Cologne, Cologne, Germany; 2 School for Child and Adolescent Cognitive Behavior Therapy (AKiP), Medical Faculty of the University of Cologne, Pohligstraße 9, 50969 Cologne, Germany; 3 Faculty of Human Sciences, University of Cologne, Cologne, Germany; 4 Department of Child and Adolescent Psychiatry/Psychotherapy, University of Ulm, Ulm, Germany; 5 Department of Child and Adolescent Psychiatry and Psychotherapy, Brandenburg Medical School Brandenburg, Neuruppin, Germany; 6 Department of Child and Adolescent Psychiatry, Neurology, Psychosomatics, and Psychotherapy, University Medical Center Rostock, Rostock, Germany; 7 Institute of Medical Statistics and Computational Biology (IMSB), Faculty of Medicine and University Hospital Cologne, University of Cologne, Cologne, Germany; 8 Department of Child and Adolescent Psychiatry and Psychotherapy, TU Dresden, Dresden, Germany; 9 Department of Child and Adolescent Psychiatry, Psychotherapy, and Psychosomatics & Research Unit Child Public Health, University Medical Center Hamburg-Eppendorf, Hamburg, Germany; 10 Department of Child and Adolescent Psychiatry and Psychotherapy, Central Institute of Mental Health, Medical Faculty Mannheim, University of Heidelberg, Mannheim, Germany

**Keywords:** Affective dysregulation; disruptive mood dysregulation disorder, Irritability, Parent management training, Cognitive behavioral therapy, Out-of-home care

## Abstract

**Background:**

The terms affective dysregulation (AD) and irritability describe transdiagnostic dimensions and are characterized by an excessive reactivity to negative emotional stimuli with an affective (anger) and a behavioral component (aggression). Due to early onset, high prevalence and persistence, as well as developmental comorbidity, AD in childhood is one of the most psychosocially impairing and cost-intensive mental health conditions. AD is especially prevalent in children in the youth welfare service. Despite continuous research, there remains a substantial need for diagnostic approaches and optimization of individualized treatment strategies in order to improve outcomes and reduce the subjective and economic burden.

**Methods:**

The ADOPT (Affective Dysregulation – Optimizing Prevention and Treatment) Consortium integrates internationally established, highly experienced and interdisciplinary research groups. The work program encompasses (a) epidemiology, including prevalence of symptoms and disorders, (b) development and evaluation of screening and assessment tools, (c) stepped care approaches for clinically useful personalized medicine, (d) evaluation of an easily accessible and cost-effective online intervention as indicated prevention (treatment effects, moderation/mediation analysis), and (e) evaluation of an intensive personalized modular outpatient treatment in a cohort of children with AD who live with their parents and in a cohort of children with AD who live in out-of-home care (treatment effects, moderation/mediation analysis).

**Discussion:**

The results will lead to significant recommendations for improving treatment within routine clinical care in two cohorts of children with AD and coexisting conditions, especially oppositional-defiant disorder, conduct disorder and disruptive mood dysregulation disorder.

**Trial registration:**

Trial registration ADOPT Online: German Clinical Trials Register (DRKS) DRKS00014963. Registered 27 June 2018.

Trial registration ADOPT Treatment: German Clinical Trials Register (DRKS) DRKS00013317. Registered 27 September 2018.

Trial registration ADOPT Institution: German Clinical Trials Register (DRKS) DRKS00014581. Registered 04 July 2018.

**Electronic supplementary material:**

The online version of this article (10.1186/s12888-019-2239-8) contains supplementary material, which is available to authorized users.

## Background

Affective dysregulation (AD) or irritability is characterized by excessive reactivity to negative emotional stimuli with an affective (anger) and a behavioral component (aggression). Accordingly, individuals with AD are overly angry or aggressive in response to provocations. Depending on the defining concept of AD, prevalence rates among children and adolescents range from 0.8 to 6.6% [1, 2]. AD or irritability is a criterion for many diagnoses in children in the 5th edition of the Diagnostic and Statistical Manual of Mental Disorders (DSM-5) [3] and the 10th revision of the International Classification of Diseases (ICD-10) [4], including mood and anxiety disorders, attention-deficit/hyperactivity disorders (ADHD), conduct disorders (CD), and oppositional defiant disorders (ODD). Given the overlap of AD symptoms with the criteria for these diagnoses, it is not surprising that high rates of AD are found in children with ODD/CD, ADHD, anxiety disorders, and mood disorders [5], as well as in children with attachment disorders and posttraumatic stress disorders. Conversely, in children with AD, high rates of these comorbid conditions can be found. AD features are most prominent in ODD [6, 7].

AD is not a diagnostic entity in the DSM-5 or ICD-10, but fits well within the framework of the National Institute of Mental Health Research Domain Criteria (RdoC) initiative [8], which investigates dimensional constructs that cut across multiple diagnoses and can be examined on multiple levels. The current RdoC include the construct of frustrative non-reward within the negative emotionality domain that encompasses AD [9]. The recently published 11th revision of the International Classification of Diseases (ICD-11) defines AD as a diagnostic additive to ODD, which can now be diagnosed as ODD with or without AD [10].

There is broad evidence that at least 30–50% of children living in institutional care and foster care (out-of-home care; OHC) have mental health problems. Indeed, several studies have shown that up to 90% of these children fulfill criteria for at least one psychiatric diagnosis [11–13]. Externalizing behavior and diagnoses, including AD symptoms, have been found to be especially prevalent in children living in OHC. Mental disorders often lead to multiple placements and to a discontinuation of youth welfare service interventions, culminating in poorer outcomes later in life [14]. An analysis employing the definition of the Child Behavior Checklist Dysregulation Profile (CBCL-DP) reported that the prevalence of AD symptoms in 10–14-year-old children in OHC in Switzerland lay at 11.1% for girls and 13.5% for boys [15].

In both preventive and psychotherapeutic contexts, behavioral parent management training (PMT) is regarded as an evidence-based treatment to decrease child externalizing behavior problems [16], including symptoms of AD. As shown by various meta-analyses, cognitive-behavioral PMT is effective in reducing child externalizing behavior problems in general [17], in children with ADHD [18], and in children with ODD and CD [16]. Analyses of mediating processes in PMT have attributed the positive treatment effects to the reduction of dysfunctional parenting [19] as well as to increases in positive parenting [20] and improvements in parent-child relationships [21].

Effects of PMT vary based on characteristics of the family and the child [22]. An example of family factors that are reported to undermine the effectiveness of PMT is family adversity, such as low socioeconomic status or single parenthood. Previously reported child factors predicting PMT outcome include the nature and severity of symptoms [23] as well as child gender [24]. The two meta-analyses of PMT reached different conclusions with respect to the moderation of interindividual differences in treatment outcome: While [22] found that economic disadvantage was the most salient moderator, favoring children with less family adversity, the more recent review by [17] did not find moderating effects either for symptom severity or for socioeconomic status.

Despite clear evidence supporting the efficacy and cost-effectiveness of PMT, interventions can be hard to access for families living in rural areas or exhibiting psychosocial adversities [17]. Practical factors which hinder parents’ attendance include lack of local availability, transportation, or childcare [16]. Book- or Internet-based, telephone-assisted self-help PMT interventions seem to be effective and easy-to-access alternatives to face-to-face PMT [25–27]. In their meta-analysis, [25] estimated the pooled standard mean difference of 11 trials of self-directed parenting interventions with no face-to-face therapist input. The examined interventions mostly comprised book- or video-based self-help programs, and three out of the 11 programs combined books with online tools. The authors reported large effect sizes (*M* = 1.01; 95% CI = 0.7–1.24) for parent-reported change in child problem behavior. However, the effects did not hold (*SMD* = 0.15) when observed child behavior was considered. Moreover, for all outcomes, the effect sizes decreased after the removal of interventions that involved regular therapist contact via telephone or Internet, suggesting that this had at least some mediating effect. In a systematic review of 11 studies, [27] identified nine different digital delivery methods (e.g., Internet, television, DVD), six of which used the Internet as the primary method. The average proportion of digitally delivered intervention completion lay at 78.3%, far outperforming the attendance rates of face-to-face PMT [27].

Our own research group in Cologne has conducted various randomized controlled trials (RCTs) on book-based and telephone-assisted self-administered PMT. One year after the intervention, positive treatment effects on child externalizing behavior problems were maintained and the number of children with externalizing behavior within the clinical range was decreased [28, 29]. A comparison between a behavioral and a nondirective guided self-help parent intervention in a clinical sample of children with externalizing disorders suggested that ODD, but not ADHD symptoms, showed greater decreases in the behavioral intervention group than in the nondirective group [30]. An analysis of mediating processes attributed this differential treatment effect to a change in parents’ dysfunctional attributions (less hostile intentions) [31], implying that this aspect should be covered in PMT.

Cognitive-behavioral PMT can thus be regarded as an evidence-based treatment to reduce child disruptive behavior across various populations, subgroups within populations, and delivery settings. It is also effective when delivered in self-administered, Internet-based formats. However, little is known about the effectiveness of similar parenting interventions in relation to child emotional problems like irritability [17, 32]. So far, PMT interventions have focused on the behavioral but not the affective component of AD. Preliminary evidence suggests that in order to meet the needs of parents with highly irritable children, regular PMT should be augmented with components focusing on emotion regulation [33]. For instance, [34] found that the presence of high levels of externalizing symptoms predicted reduced efforts by parents to engage in emotion regulation coaching with their child. This suggests that there might be an especially harmful interplay between child temperamental and behavioral characteristics and parenting behavior in children with AD with additional externalizing problems. Parents of children with AD therefore need to be especially trained in promoting their child’s emotion regulation. As parenting a child with AD can be emotionally challenging, and negative affect makes hostile and coercive reactions of parents more likely [35], parents’ own emotion regulation capacities should be enhanced as well. The model of mindful parenting might be a helpful framework to support emotional awareness and regulation of parent and child by increasing parents’ full attention and nonjudgmental acceptance of the child, and by supporting self-regulation in the parenting relationship [36]. Recent findings suggest that mindful parenting increases parents’ responsiveness [37] and regulation of negative affect [38]. Heightened parental mindfulness has been found to lead to a decrease in parental stress and child behavior problems [39, 40].

To the best of our knowledge, there are no previous or ongoing studies evaluating extended self-help PMT for children with AD, which cover child and parent emotion regulation in addition to regular PMT topics. As severely affected children with AD often live in families suffering from multiple adversities, such specialized treatments should address the specific needs of this population of parents. Easy-to-assess Internet-based interventions seem to be especially promising for highly burdened parents.

Besides PMT, cognitive behavior therapy (CBT) delivered as (child-centered) psychotherapy has also been shown to be effective for children with externalizing disorders, ADHD, and ODD/CD [16, 41, 42]. We identified four meta-analyses on the effects of psychosocial interventions for children with AD/anger [41, 43–45]. Sukhodolsky et al. [41] identified 21 published and 19 unpublished reports on (patient-based) CBT for anger-related problems in children and adolescents and calculated an overall mean effect size of Cohen’s *d* = 0.67 (anger *d* = 0.72; aggression *d* = 0.63). The differential effects of skills training, problem solving, affective education, and multimodal interventions were variable, although generally also in the medium range. A meta-analysis including 60 studies found that school-based anger management interventions were effective for outcomes including anger, aggression, and loss of self-control (overall effect sizes: *d* = 0.27 [43], *d* = 0.31 [44]). Another meta-analysis of anger management training interventions reported an overall moderate effect size of *d* = 0.61, although this study was limited only to students with special educational needs [45]. Effects of classroom-based and teacher-involved interventions are also documented for patients with aggressive behavior (ODD/CD) and for ADHD. Universal prevention was found to be effective in reducing aggressive behavior in a meta-analysis conducted by the National Institute for Health and Care Excellence (four included studies; *d* = 0.43) [16].

Currently, several studies are planned or underway, which aim to investigate the efficacy of interventions to reduce anger, emotional dysregulation or irritability in children using dialectical behavior therapy [NCT01299740], an interactive biofeedback video game to regulate and gain emotional control [NCT01551732], or CBT [NCT01965184].

Most of the studies reported in the meta-analyses and all of the ongoing trials consist of child-based treatments that use standard group format programs and no personalized approach. This is also the case for the transdiagnostic approach to CBT for anger/irritability and aggression, which combines interventions that aim at (a) regulating excessive anger, (b) learning social problem-solving strategies, and (c) developing social skills alternatives to aggressive behaviors [32, 46]. Group-based interventions are less expensive, but may be less effective than individual treatment approaches [47, 48].

Additionally, most traditional evidence-based CBT programs focus on one disorder (or a small cluster of related disorders), making it difficult for clinicians to address heterogeneous caseloads, client comorbidities, and changes in clinical presentation during therapy. Training for each single-focus therapy can be time-consuming and costly, and research participants in RCTs are typically treated for one disorder (whereas in clinical practice, comorbidity is common).

Due to the diversity of symptoms of AD and associated disorders, a more flexible modular approach to therapy for children is needed. Clinicians should be guided by an evidence-based algorithm to tailor treatment to each individual’s characteristics and needs. This is especially true for high-risk populations like children in OHC, who show high rates of adverse childhood experiences, which is a strong predictor of psychopathology. Psychotherapeutic approaches should comprise modules that can be organized in a flexible manner. Moreover, these modules should be based on cognitive-behavioral and skills-based approaches for which meta-analyses have shown the best evidence of effectiveness for these disorders associated with AD, anger, and irritability. Furthermore, efforts should be undertaken to identify frequently used common elements of evidence-based practice for children.

Personalized individual modular treatment approaches are rare [49], because individualized treatment packages address specific deficits, for instance in anger control. One of the few empirically tested personalized modular approaches is the Modular Approach to Therapy for Children with Anxiety, Depression, Trauma, or Conduct Problems (MATCH-ADTC), which targets youth who have any one or any combination of these problems [50]. An RCT (*n* = 174) found that MATCH-ADTC was more effective than standard (single-focus) evidence-based therapies and usual care, with effect sizes of 0.59 to 0.71 for primary outcome variables [51]. Our own research indicates that personalized modular CBT of the patient improves ODD symptoms (including irritability) and aggressive behavior [48, 52], and personalized modular parent training improves ODD symptoms in children with ADHD [53].

Evidence-based interventions to improve mental health are particularly lacking for children living in OHC, who have high rates of adverse childhood experiences [54]. According to the German 13th Child and Youth Report and a statement from the German government, effective trauma-sensitive interventions are required in this population [55]. Their needs are under-addressed in both the youth welfare system and the health care system. Therefore, an early intervention approach using evidence-based CBT interventions to reduce AD symptoms is urgently needed, and has not yet been investigated. Given the variability and complexity of the clinical picture of AD in combination with comorbid conditions, a modular approach with a stepped care design is necessary, which tailors evidence-based interventions to fit patient needs.

Most previous studies used a group-based standardized treatment approach, both in parent-based interventions and in child-based cognitive-behavioral interventions. Only a small number of studies have combined the effective components of child-based, parent-based, and teacher-based interventions. Assessments of individual modular approaches are rare. The only personalized modular approaches either have a broader scope and include children with internalizing symptoms (like MATCH-ADTC) [50] or focus on specific disorders such as ADHD or ODD/CD [48, 53]. The efficacy of an intensive personalized modular approach including modules on CBT and PMT in comparison to treatment as usual (TAU) has not been evaluated [42, 49]. The effects on specific measures of AD and moderating as well as mediating factors are largely unknown.

Almost none of the studies published to date, or which are currently being processed, assessed the effects of an intervention within a stepped care approach (except 53). Symptoms of AD seem to vary broadly and differ in intensity. Thus, a stepped care approach that includes interventions with increasing intensity could meet the individual needs of patients and families that arise from different forms of AD.

ADOPT Online, ADOPT Treatment and ADOPT Institution are all part of the research consortium ADOPT (Affective Dysregulation – Optimizing Prevention and Treatment; https://www.adopt-studie.de). The goals of the umbrella project are to (a) investigate the epidemiology of AD including prevalence of symptoms and disorders, and biological and psychosocial risk and protective factors (ADOPT Epidemiology; ADOPT Neurobiology); (b) develop and evaluate screening and assessment tools (ADOPT Epidemiology; ADOPT Treatment); and (c) develop and evaluate treatment approaches for clinically useful personalized medicine in two cohorts of children with AD (ADOPT Online; ADOPT Treatment; ADOPT Institution; see detailed description below).

The primary objective of ADOPT Online is to assess the overall efficacy of a PMT program that can be accessed over the Internet (Online Parent Self-Help of Affective Dysregulation and coexisting disorders; ADOPT Online). In general, self-help or online PMT have proven to be effective in reducing child behavior problems. However, they might only be capable of reaching a subpopulation of parents [56], e.g., those who are better educated, less burdened, or whose children are less affected. Self-help interventions can thus serve as a time- and cost-effective first step of intervention that can reduce symptoms in a subpopulation [25, 49].

In the framework of a stepped care approach, the primary objective of ADOPT Treatment is to assess the overall efficacy of a personalized modular outpatient treatment of AD and coexisting disorders (Therapy for Optimizing Affective Regulation in children; THOPAS) in children with substantial residual symptoms of AD after parents have received Internet-based PMT (in ADOPT Online).

The primary objective of ADOPT Institution is to assess the overall efficacy of THOPAS in 8–12-year-old children living in OHC. These children are expected to have higher rates of AD than children who live with their natural parents. It is assumed that the treatment effects on AD in children in OHC may differ from the effects on children living with their parents, due to a higher symptom load and more adverse childhood experiences in the former group. Furthermore, professional educational staff are already involved in institutional care. Online guidance for effective parenting cannot be considered as an appropriate intervention, since the staff of the youth welfare service will already possess knowledge about positive parenting. Therefore, there was a sound rationale for a specific study design for this population, which provides direct, intensive treatment.

ADOPT Treatment and ADOPT Institution thus intend to fill some of the aforementioned research gaps by assessing the effects of THOPAS, either in a sample of children who live with their parents or in a sample of children living in OHC. Treatment strategies for multiple problems are organized into self-contained modules that can be used multiple times or not at all, and can be combined as needed; clinical decision-making flowcharts give guidance about which modules to use and when to use them for a particular patient [49]. Moreover, this intensive personalized intervention will be applied within a stepped care approach only in those cases in which a less intensive and less expensive self-help online intervention has proven to be insufficiently effective (see ADOPT Online).

In all three ADOPT subprojects, the following principal research questions will be addressed:

1. What is the overall efficacy of ADOPT Online and of THOPAS (ADOPT Treatment and ADOPT Institution) on AD and comorbid conditions, functional impairment and psychological well-being in comparison to TAU in children (living with their parents or in OHC) aged 8;0 to 12;11? What is the clinical significance of the symptom change in terms of normalization rates (in comparison to a control group of participants without AD, defined at T1) or reliable changes (RCI) [57]?
2. How feasible is ADOPT Online and THOPAS and how satisfied are parents/caregivers with the intervention?
3. Can specific psychopathological profiles be identified (e.g., AD with high ADHD compared to AD with low ADHD) that moderate/predict treatment outcome?
4. Which other moderators/predictors (e.g., gender, age, parental mental health, adverse childhood experiences) can be identified to predict treatment outcome?
5. Do the theoretically expected treatment mechanisms work?
6. What is the stability of the treatment outcome?
  Additionally, the following research question will be addressed in ADOPT Treatment and ADOPT Institution:
7. Can specific combinations of treatment modules be identified (e.g. anger control training plus parent/caregiver management training to reduce dysfunctional parenting/caregiving; trauma therapy to reduce trauma-specific symptoms) that predict treatment outcome?
8. Do children in OHC respond to treatment similarly to children who live with their natural parents, or can specific moderators of treatment response be identified?
  Furthermore, the following research questions will be addressed in the other two ADOPT subprojects (ADOPT Epidemiology and ADOPT Neurobiology), which are not described comprehensively in the present manuscript:
9. What are the prevalence rates of AD in a community sample and what are the comorbidity rates in children with AD? (ADOPT Epidemiology)
10. What are the psychosocial risk and protective factors for AD and comorbid conditions in children with AD, and how are AD and comorbid conditions associated with well-being in children? (ADOPT Epidemiology)
11. What (neuronal) alterations of affective processing, reward processing, cognitive control and attentional functions can be identified in children with AD? (ADOPT Neurobiology)
12. What are the neuropsychological and/or neurobiological predictors of response to personalized psychotherapeutic treatment in children with AD? (ADOPT Neurobiology)
13. What are the relations between neurobiological markers, neuropsychological measures, real-life behavior, retrospective parental ratings and self-ratings of AD? (ADOPT Neurobiology)

## Methods

### Trial design

**Fig. 1:**
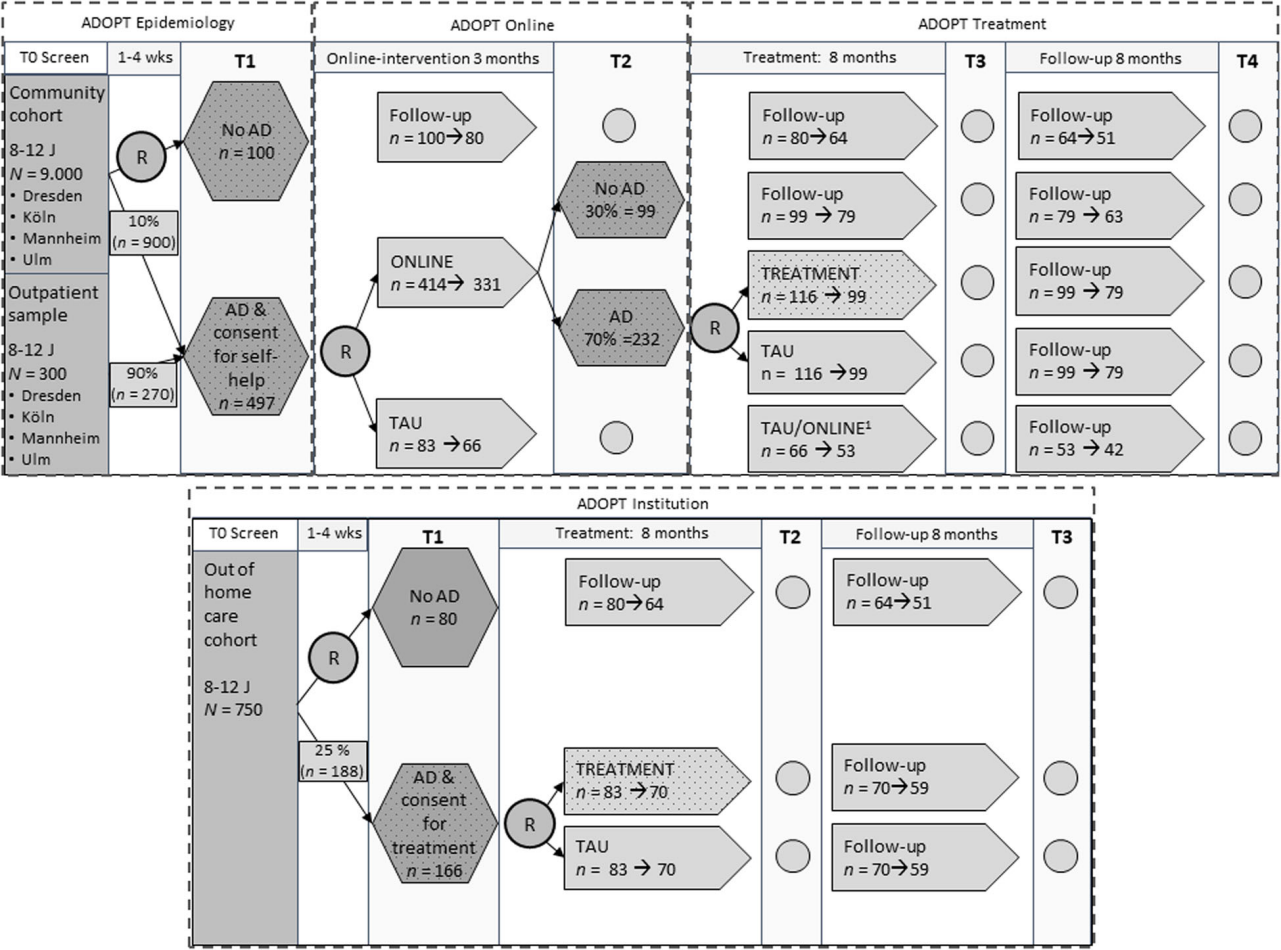
Study design of the ADOPT consortium including ADOPT Epidemiology, ADOPT Online, ADOPT Treatment, ADOPT Institution as well as ADOPT Neurobiology. Percentages indicate expected proportions of children with or without affective dysregulation. Dotted boxes indicate subsamples used in ADOPT Neurobiology. T1 to T4 = assessment points; R = randomisation; AD = affective dysregulation; ONLINE = web-based parent training; TAU = treatment as usual; TREATMENT = individualized cognitive behavioural psychotherapy of child, parents, teachers; *n* = planned/estimated sample size. ^1^ Patients / Families can choose between continuation of TAU and participation in Online

Figure 1 shows the overall design of the whole ADOPT consortium. Sample recruitment in the community cohort for ADOPT Online and ADOPT Treatment will be executed by another ADOPT subproject (ADOPT Epidemiology) and the initial screening will be performed in a community sample. Sample recruitment in the outpatient sample in ADOPT Online and ADOPT Treatment will be performed by the study centers. The screening for AD in ADOPT Institution will take place in children who live in OHC.

Children will be selected for inclusion via a screening instrument (Diagnostic Tool for Affective Dysregulation in Children – Screening Questionnaire; DADYS-Screen) [Görtz-Dorten et al., 2018, unpublished manuscript; Otto et al., 2018, unpublished manuscript] which will be developed by ADOPT Epidemiology. In addition, children who show symptoms of AD according to clinical judgment (outpatient sample) will also be included. ADOPT Epidemiology will draw a community sample to screen for children with AD. In total, data of *N* = 13,000 children aged 8 to 12 years will be collected across four German Cities (Cologne, Dresden, Mannheim, Ulm). The participants will be recruited through the residents’ registration offices of the four cities. In order to account for an estimated dropout rate of 70–75%, the contact details of 78,000 children will be requested from the registration offices. Based on screening data of the ADOPT project, groups of children with high vs. low AD will be defined using a cut-off which will be determined by means of a statistical case definition (10th percentile). Additionally, *n* = 100 cases without AD will be randomly drawn from the community sample and asked about their willingness for ongoing participation in the ADOPT study (No AD control group). The ADOPT Epidemiology study office will inform the study centers (see section “Study Setting”) about screening-positive cases as well as participants of the control group. In the outpatient sample, children will be enrolled by clinicians. The study centers will invite the participants and their parents to further participate in the study from T1 onwards.

At T1, symptoms of AD will be more intensively evaluated by clinicians’ ratings based on parent interviews conducted with the outcome measure for AD (Diagnostic Tool for Affective Dysregulation in Children – Parent Interview; DADYS-PI) (Görtz-Dorten et al., 2018, unpublished manuscript), which will be developed in ADOPT Epidemiology in cooperation with ADOPT Treatment. Exclusion criteria (intelligence quotient < 80; mental disorder other than comorbid conditions is the primary disorder and the main cause of AD (e.g., autism spectrum disorder); current or planned intensive behavioral therapy (ADOPT Online)/intensive psychotherapy or PMT (ADOPT Treatment/ADOPT Institution) on a weekly/biweekly basis) will also be assessed at T1/T2 (ADOPT Treatment), along with the residence of the child (ADOPT Online/ADOPT Treatment: with natural or adoptive parents; ADOPT Institution: residential care or foster care). Additionally, two healthy control groups (No AD; in ADOPT Online/ADOPT Treatment and in ADOPT Institution) will be identified via the screening and will be pursued from T1-T4 (ADOPT Online/ADOPT Treatment) and from T1-T3 (ADOPT Institution).

In ADOPT Online, children selected from the community sample and from the outpatient sample of the study centers will be randomized to TAU or ADOPT Online. Children will be reassessed after parents have had access to ADOPT Online for 3 months (T2). Families of the community cohort randomized to TAU will gain access to ADOPT Online after T2. Families of the outpatient sample randomized to TAU will be offered the choice between continuing TAU and participating in the online intervention (ADOPT Online) after T2. From T3 to T4, ADOPT Online will also pursue those children who were in the No AD group from the beginning as well as those who showed no residual AD at T2. For detailed information see Additional file 1: Trial registration ADOPT Online.

In ADOPT Treatment, children with substantial residual symptoms of AD after ADOPT Online, as rated by the clinician on the outcome measure for AD based on parent interview (DADYS-PI), will be included in ADOPT Treatment. The patients will be randomized to TAU or the psychotherapy intervention, which will comprise a modular, individual behavioral approach based on THOPAS [Görtz-Dorten & Döpfner, 2018, unpublished manuscript]. For detailed information see Additional file 2: Trial registration ADOPT Treatment.

In ADOPT Institution, a total of *n* = 750 children in OHC will be screened via a short screening instrument for caregivers (DADYS-Screen) (Görtz-Dorten et al., 2018, unpublished manuscript; Otto et al., 2018, unpublished manuscript) at T0. Children with AD as rated by the clinician on the outcome measure for AD (DADYS-PI) based on parent interview will be included in ADOPT Institution. The patients will be randomized to TAU or the psychotherapy intervention with THOPAS. For detailed information see Additional file 3: Trial registration ADOPT Institution.

### Study setting

ADOPT Online and ADOPT Treatment are multisite trials with five study centers located in four cities in Germany (Cologne, Dresden, Mannheim, Ulm). If required, additional centers will be included. ADOPT Institution is a multisite trial with six study centers located in five cities in Germany (Cologne, Dresden, Mannheim, Ulm, Neuruppin). The coordinating center for ADOPT Online is the Department of Special Education at the Faculty of Human Sciences at the University of Cologne. The coordinating center for ADOPT Treatment is the Department of Child and Adolescent Psychiatry, Psychosomatics and Psychotherapy, Medical Faculty at the University of Cologne. The coordinating center for ADOPT Institution is the Department of Child and Adolescent Psychiatry/ Psychotherapy at the University of Ulm. Patients will be treated locally at the respective study centers, with the exception of ADOPT Online, which will be provided centrally online. Responsibility for the coordination of the whole ADOPT consortium and data management is held by the Department of Child and Adolescent Psychiatry, Psychosomatics and Psychotherapy, Medical Faculty of the University of Cologne. Responsibility for monitoring lies with the Clinical Trials Center Cologne. The Institute of Medical Statistics and Computational Biology at the University Hospital Cologne (IMSB) is responsible for statistical analysis.

### Inclusion and exclusion criteria

Participants will be included if they meet the following inclusion criteria: (i) child age: 8;0 to 12;11 years at T1; (ii) child is resident with at least one natural parent/adoptive parent (ADOPT Online/ADOPT Treatment) or (ii) child lives in residential care/foster care (ADOPT Institution); (iii) clinician-rated AD symptoms of the child (DADYS-PI) based on parent interview > cut-off at T1 (ADOPT Online/ADOPT Institution)/T2 (ADOPT Treatment); (iv) willingness and ability of patient and parents/guardians to participate in the intervention.

The following exclusion criteria will be applied and evaluated at T1 (for ADOPT Treatment): (i) child’s intelligence is below average based on clinical evaluation/child attends school for children with intellectual disabilities; (ii) mental disorder other than comorbid conditions is the primary disorder and the main cause of AD (e.g., autism spectrum disorder); (iii) current or planned intensive behavioral therapy (ADOPT Online)/psychotherapy (ADOPT Treatment and ADOPT Institution) or behavioral PMT on a weekly/biweekly basis.

In ADOPT Treatment and ADOPT Institution, therapists administering THOPAS will have a university degree in psychology or education and will be trained in behavioral psychotherapy.

### Allocation

Patients fulfilling the eligibility criteria at T1 will be centrally registered and will be randomly assigned to the treatment groups (ADOPT Online or TAU, allocation ratio 5:1; THOPAS or TAU, allocation ratio 1:1) according to permuted blocks of varying length. Randomization will be stratified by recruitment process (ADOPT Online and ADOPT Treatment), gender and study site (i.e. 20 strata altogether for ADOPT Online/ADOPT Treatment, 12 strata altogether for ADOPT Institution) and implemented as a central 24–7 Internet service (ALEA, FormsVision BV, Abcoude, NL) operated by local authorized study staff. Treatment assignments will be displayed on screen and delivered by e-mail. The randomization service will be maintained by the IMSB, University of Cologne.

### Interventions

#### ADOPT online

The material for ADOPT Online is based on an established web-based ADHD parent-trainer [58], which is adapted from evidence-based, print-based parent self-help programs for reducing child externalizing behavior problems [26, 32, 59]. The content and dosage of the self-help programs have been evaluated previously [29, 30, 60]. While the original version of the parent-trainer focuses on changing the antecedents and consequences of problem behavior and thus aims at changing the behavioral component of AD, the modified online training combines these interventions with aspects from the mindful parenting model and with components from schema therapy.

The concept of effective parenting in the ADOPT Online parent training combines parental control with positive attention and support of the child’s emotion regulation to strengthen the child’s self-efficacy. ADOPT Online provides parents with access to three modules; the contents of these modules are interconnected, and parents can work through them in a flexible manner. In the first module (“Strengthening your child”), parents are instructed to support their child to regulate aversive emotions using a 5-step plan that involves mindfulness-based techniques to perceive and identify feelings [61], taking a self-compassionate perspective [62], and using cognitive and behavioral strategies to regulate emotions [63–65]. Additionally, the module leads parents to analyze individual problems with their child using videotaped examples (e.g., permanently bad mood, outbursts of fury) in a step-by-step manner. Effective strategies to change behavior problems are presented (e.g., ways to improve parent-child interactions, defining clear rules, applying appropriate positive and negative consequences) [66]. Parents are guided to use these strategies on their child and to document the outcome. In the second module (“Strengthening yourself”), parents are instructed to apply the 5-step plan to their own emotions. This module helps parents to deal with their own negative emotions, which might occur as a reaction to the child’s AD. Furthermore, parents are instructed to improve the structures in their everyday life and to activate their own resources. In line with schema therapy, parents are guided to identify dysfunctional behavior that leads to strong negative emotions [67, 68]. The third module (“Feelings: Worth Knowing”) incorporates psychoeducation on AD (e.g., AD as a disorder; the role of emotions; the development of emotion regulation strategies) [63, 64, 69]. The ADOPT Online training is illustrated with sketches and short films to support social learning without direct therapist contact.

We have chosen a three-month (= 12 weeks) duration for ADOPT Online in order to provide sufficient time to achieve a satisfactory degree of change for those parents who use the material intensively [70]. Moreover, parents who fail to use the material within this period of time are unlikely to begin using it after 3 months; for these parents, and for children with residual AD symptoms, the next step of the stepped care approach then needs to be addressed.

The entire material will be available from the beginning of the three-month intervention period. During this three-month period, participants will receive reminders and reinforcement via automatically sent e-mails. Information about the use of the online tool will be collected from the system after participating parents have provided informed consent. The online material will be hosted by a professional service bound to data safety and security by strict contracts that permit only the study personnel to use any data produced by the participants.

#### THOPAS

THOPAS consists of 10 CBT modules that include child-based interventions, parent training interventions, and teacher coaching. The 10 modules comprise interventions to activate resources and build a positive therapeutic relationship (module 1), interventions to strengthen positive parent-child interactions (module 2) and to reduce dysfunctional parenting (module 3), interventions to strengthen positive teacher-child interactions (module 4) and to reduce dysfunctional teacher behavior towards the child (module 5), anger control training and training of emotion regulation (module 6), empathy training (module 7), social problem solving and social skills training (module 8), organizational skills training (module 9), and coping with trauma and negative life events (module 10).

In the parent-training and teacher-coaching modules (2 to 5), target problems of the child are identified, along with the child’s competencies and the coercive interaction process. The problem of maintaining social interactions is addressed in the modification of social interactions module, in which social rules are developed in order to reduce the target problems of the child. This module includes how to communicate effective commands, how to coach the child in social problem situations, methods for rewarding the child (e.g., token systems) when the child shows prosocial behavior, or appropriate methods of punishment (e.g., time-out) when the child shows aggressive behavior. Additionally, these sessions aim at identifying and modifying parents’ and teachers’ dysfunctional thoughts about the child, as well as their own aggressive behavior, impulse control, and conflict management.

The goals of the child-based interventions (modules 6 to 9) are to identify and reduce anger-inducing cognitions (*Anger thoughts*) and basic dysfunctional ideas (*Thought traps*; for example “I have to be better than everyone”), and to develop empathy (*Take another person’s perspective*). The anger control training addresses impulse control (*Control your anger*), while the social problem-solving and social skills training aims at helping patients to develop and evaluate alternative solutions in a problem situation, as well as to train them in skillful non-verbal and verbal behavior, including role-play, video feedback, and role-reversal. Organizational skills training helps children to organize their daily tasks at home and at school. In module 10, traumatic experiences and negative life events are identified together with the child; coping thoughts and a written narrative are developed as the basis for a gradual exposure. The child-centered interventions also include guidance for parents/teachers to support the child’s behavioral change at home/school.

The interventions in module 1 and modules 6 to 10 will be supported by a smartphone app which has been developed for child psychotherapy. The app will be installed onto the child’s smartphone or onto a therapy smartphone that the child can take home to keep for the duration of the therapy. With the help of the app, children will be able to record therapy-relevant situations and emotions and to transfer coping strategies from therapy sessions to daily life. Self-management, therapeutic homework assignments, and interventions in the real-life setting (*Can you manage that in the real world?*) with self-reinforcement are added to each of these modules.

The parent training interventions (modules 2 and 3) are based on the evidence-based Treatment Program for Hyperkinetic and Oppositional Problem Behavior (THOP) [66], which is adapted from evidence-based international treatment manuals, in particular Barkley’s Defiant Children [71] and Helping the Non-compliant Child [72]. The teacher coaching (modules 4 and 5) is also based on the THOP program, as well as the newly developed German-language School-based Coaching for Teachers of Children with Disruptive Behavior Problems (SCEP) [73], which is adapted from evidence-based international treatment manuals, in particular [74]. The child-centered interventions in modules 6–8 are based on evidence-based German treatment programs – the Treatment Program for Children with Aggressive Behavior Problems (THAV) [75], and the Social Computer-assisted Training for Children with Aggressive Behavior Problems (ScouT) [47] – which are adapted from evidence-based international treatment manuals, in particular the Coping Power Program [76] and the Cognitive-Behavioral Therapy for Anger and Aggression in Children [46]. Module 9 is adapted from the Organizational Skills Training for Children with ADHD manual [77]. Module 10 is adapted from Trauma-Focused Cognitive-Behavioral Therapy for Traumatized Children (TF-CBT) [78].

The whole treatment encompasses a total of 24 sessions. For each patient, a personalized treatment plan will be developed by combining the treatment modules according to the patient’s specific problems and problem-maintaining factors, as assessed prior to treatment (by rating scales, observational measures, and psychological tests). The indication for each module will be operationalized and the decision for the personalized combination of the modules will be documented. The CBT modules will be combined according to the specific problems of the child (e.g. lack of problem-solving skills or social skills, lack of anger control or emotional regulation, lack of empathy, lack of organizational skills, experience of traumatic events), the specific problem-maintaining factors in the family (lack of parenting skills, lack of positive parent-child interaction), or the specific problem-maintaining factors at school (dysfunctional teacher-child interactions, lack of positive teacher-child interaction). Clinical decision-making flowcharts will be used, similar to those developed in MATCH-ADTC [50] and the German treatment programs THAV [75] and THOP [66]. In children with severe ADHD, the clarification of an indication for psychopharmacological treatment by an external physician will be recommended. Whenever pharmacological treatment is recommended according to current treatment guidelines, these interventions will be decided upon and fully carried out by the clinical physicians of the respective participating patient, independent from the study personnel.

#### Treatment as usual (TAU)

The control intervention includes TAU with an intervention duration per patient of 12 (ADOPT Online) or 32 (ADOPT Treatment/ADOPT Institution) weeks. No treatment condition is deemed unacceptable on ethical grounds. TAU as control condition provides information about the additional benefit compared to usual care. As there is no gold standard in the treatment of AD, an active treatment as a comparator is not feasible. Within TAU, all psychosocial, psychotherapeutic and pharmacological interventions will be documented.

### Concomitant care

Children with psychotropic medication and continued symptoms of AD under medication at pre-treatment assessment (T1/T2) will be included in the study. In ADOPT Treatment and ADOPT Institution, children with comorbid ADHD may be referred to a child and adolescent psychiatrist to evaluate the indication for additional pharmacological treatment. Decisions on possible pharmacological treatment will lie with the attending physician and will not be altered due to the study. Documentation of pharmacological treatment will be limited to the question (answered by the parents) of whether the child is receiving any kind of psychotropic medication and if so, for the treatment of which symptoms (ADHD/AD or other). Other intensive (behavioral) psychotherapies or PMT (on a weekly/biweekly basis) are not permitted in the experimental conditions.

### Treatment fidelity

In ADOPT Online, there will be no therapist contact with the participating parents. As the online intervention will be the same for all participants, no strategies to improve treatment fidelity are necessary. In ADOPT Treatment and ADOPT Institution, treatment fidelity will be assured by (i) training in manualized treatment procedures, (ii) a structured protocol completed by therapists after each session, and (iii) supervision of behavior therapy by senior supervisors, either face to face or by telephone. Behavior therapies will be supervised after every four treatment sessions, including a review of at least two videotaped sessions.

### Participant timeline

For ADOPT Online and ADOPT Treatment, the individual study duration per patient is 19 months. Measurements will take place according to a specified schedule. Immediately after the initial investigation (T0, screening), the first measurement (T1) will occur, which involves an examination of inclusion and exclusion criteria. For parents of patients randomized to the experimental group at T1, ADOPT Online will then be carried out over a period of 12 weeks. Measurement T2 will take place 13 weeks after T1. Families that were randomized to TAU after T1 will be offered ADOPT Online at T2. Non-responders to ADOPT Online at T2 (significant symptoms of AD at T2 as rated by the clinician based on parent interview) will subsequently be randomized to either THOPAS or TAU, which will each be carried out over a period of 32 weeks. T3 will occur 32 weeks after T2. The follow-up measurement T4 will take place 32 weeks after T3. Thus, for all patients in ADOPT Online, there will be four study visits (T1-T4). For non-responders to ADOPT Online, who continue treatment in ADOPT Treatment, there will be 24 additional study visits (24 behavior therapy sessions including one intermediate measurement).

For ADOPT Institution, the individual study duration per patient is 16 months. Measurements will take place according to a specified schedule. Immediately after the initial investigation (T0, screening), the first measurement (T1) will occur, which involves an examination of inclusion and exclusion criteria. Children with AD will subsequently be randomized to either THOPAS or TAU, which will each be carried out over a period of 32 weeks. T2 will take place 32 weeks after T1. The follow-up measurement T4 will be conducted 32 weeks after T2. Thus, for all patients in ADOPT Institution, there will be three study visits (T1-T3). For children randomized to THOPAS, there will be 24 additional study visits (24 behavior therapy sessions including 1 intermediate measurement).

At the main assessment points (T1-T3/T4), the primary outcome (blinded clinician-rated AD symptoms based on parent interview) and the secondary outcomes will be assessed. In ADOPT Online, information about the use of the online tool will be collected after participating parents have provided informed consent. The intermediate assessments at T1b (ADOPT Institution) and T2b (ADOPT Treatment) will only include parent- and child-report questionnaires, and no clinical interviews. During T1b/T2b, potential treatment mediators and predictors and short forms of the parent-rated AD symptoms will be assessed. In addition, the therapist in ADOPT Treatment/ADOPT Institution will complete a questionnaire to measure adherence and treatment integrity at regular intervals during the treatment.

### Informants

The following informants will be considered for assessment: unblinded clinician, blinded clinician, parent/caregiver, patient, and teacher.

The unblinded clinician will be a member of the project staff who is involved in diagnostics or in therapy (but not in the family being assessed). He/she may be aware of the treatment condition and the time of the assessment, although efforts will be undertaken to blind the raters. The blinded clinician will also be a member of the project staff, and will be aware of neither the treatment condition nor the time of the assessment. To ensure blindness of the clinical rating of the primary outcome, the parent interviews will be videotaped and subsequently rated. Any information in the video material that may disclose the treatment condition or the measurement point (e.g., T1, T2, T3, T4) will be hidden for the rating. The parent may be the biological parent or the guardian of the child, and will be involved in the treatment. The caregiver may be an educator in residential care or a foster parent. The patient is the child participating in the study/treatment. The teacher is the child’s schoolteacher, preferably the class teacher with the main responsibility for the child’s school routine.

### Outcomes

#### Primary outcome

The primary efficacy outcome is the blinded clinician-rated AD symptom score of the child based on parent/caregiver interview at T1-T4. To date, there is a lack of validated measures on AD [6]. Therefore, the DADYS-PI will be developed by another subproject of the ADOPT consortium (ADOPT Epidemiology, in cooperation with ADOPT Treatment) before the start of the trial, based on a pre-version of DADYS-PI which will be psychometrically analyzed in a clinical sample. The pre-version includes items based on the Emotion Regulation Checklist (ERC) [79], the Diagnosis Checklist for Disruptive Behavior Disorder from the German Diagnostic System for Mental Disorders in Children and Adolescents (DISYPS-III) [80], the Affective Reactivity Index (ARI) [81], the Parent Proxy Scale [82], and the Pediatric Anger Scale from the Patient-Reported Outcomes Measurement Information System [83].

Blinded clinician ratings based on parent/caregiver interviews will be used as the primary outcome, since these ratings should be less biased compared to parent ratings and self-ratings of the child. The items of DADYS-PI are scored from 0 (not present) to 3 (very strong). These items are added up to a total score, representing the extent of AD symptoms of the child.

#### Secondary outcomes

The secondary endpoints are summary scores/scale scores of the respective items. The secondary outcomes listed below will be evaluated at T1-T4, unless otherwise noted.

The secondary outcomes are (1) psychosocial impairment of the child due to AD symptoms in blinded clinical rating as well as patient, parent/caregiver, and teacher rating, (2) patient-, parent/caregiver-, teacher- and clinician-rated AD symptoms of the child, (3) patient-, parent/caregiver- and teacher-rated symptoms of ADHD and ODD/CD, (4) other comorbid conditions (e.g., anxiety, depression) assessed by parent/caregiver ratings and teacher ratings, (5) psychological well-being in patient and parent/caregiver rating, and (6) parent / caregiver satisfaction with the treatment.

Psychosocial impairment of the patient due to AD symptoms will be measured by the functional impairment scale of the new version of the DISYPS-III [80]. The items are based on the definition of functional impairment in DSM-5 and assess social impairment in relationships with adults and other children, and impairment in academic functioning. The reliability and validity of the scale have already been established in German samples [80]. The items will be integrated into the clinical interview (DADYS-PI) and into the questionnaires for patient, parent/caregiver, and teacher rating (DADYS questionnaires).

Patient-, parent/caregiver- and teacher-rated AD symptoms of the child will be measured by newly developed questionnaires (DADYS questionnaires). Additionally, there will be a clinical rating of AD symptoms of the child based on a clinical interview with the child (DADYS-CI). The instruments will be developed by another subproject of the ADOPT consortium (ADOPT Epidemiology, in cooperation with ADOPT Treatment) before the start of the trial, based on a pre-version of the DADYS questionnaires and DADYS interviews, which will be psychometrically analyzed in a clinical sample. The DADYS instruments include items based on the ERC [79], FBB-SSV/SBB-SSV from the DISYPS-III [80], ARI [81], the Parent Proxy Scale [82] and the PROMIS Pediatric Anger Scale [83].

Comorbid symptoms of ADHD and ODD will be assessed in patient and parent/caregiver rating using the respective rating scales (Self-Rating Scale for ADHD, SBB-ADHS; Self-Rating Scale for ODD and CD, SBB-SSV; Rating Scale for ADHD, FBB-ADHS; Rating Scale for ODD and CD, FBB-SSV) based on DSM-5 criteria within the DISYPS-III [80]; reliability and validity as well as sensitivity to change of these scales has already been proven in German samples [80]. Teachers will rate comorbid symptoms of ADHD and ODD using selected items of a screening questionnaire (Rating Scale for Screening; FBB-Screen) of the DISYPS-III [80].

Further comorbidities will be assessed in parent/caregiver and teacher rating with the German version of the Child Behavior Checklist (CBCL/6-18R) [84] and the Teacher Report Form (TRF/6-18R) [85]. Three subscales will be used (Anxious/depressed, Attention problems, Aggressive behavior).

Child symptoms of attachment disorders/posttraumatic stress disorders will be assessed in parent/caregiver rating using the respective scales of the DISYPS-III (Rating scale for attachment disorders, FBB-BIST; Rating scale for trauma- and stress-related disorders, FBB-TBS) based on DSM-5 criteria [80].

The KIDSCREEN questionnaires [86] measure subjective health and well-being of children and adolescents as rated by patients (KIDSCREEN-10-Index) and parents/caregivers (KIDSCREEN-27).

For the assessment of satisfaction with the interventions at T2 (ADOPT Online) or at T3 (ADOPT Treatment), specific parent/caregiver satisfaction rating scales have been developed, based on the Client Satisfaction Questionnaire (CSQ) [87].

#### Predictors/moderators of treatment outcome on AD symptoms and impairment

As predictors/moderators of treatment outcome on AD symptoms and impairment, the following variables will be analyzed: (1) gender, (2) age of patients and parents/foster parents, (3) recruitment process (community cohort vs. outpatient sample; in ADOPT Online/ADOPT Treatment); (4) chronicity of AD symptoms, (5) severity of AD symptoms, (6) severity of comorbid symptoms, (7) AD symptoms and other psychopathology of the participating parent/foster parent, (8) positive and negative parenting practices, (9) receipt of social welfare assistance by the family, (10) socioeconomic status of the family/foster family, (11) early childhood neglect of the patient, (12) adverse childhood experiences of the patient, (13) family climate according to the child’s perception, (14) social support and (15) personal resources of the child. These parameters will be assessed at T1 and/or at T2. Additionally, (16) the profile of usage of the online tool (ADOPT Online) as well as (17) the implementation of treatment modules (ADOPT Treatment/ADOPT Institution) will be assessed and analyzed as predictors of treatment outcome in the intervention groups, along with (18) treatment adherence and (19) treatment fidelity in ADOPT Treatment/ADOPT Institution, both rated by the therapist, (20) the current mood of the patient (measured with the momentary assessment function of the therapy app), and (21) behavior, thoughts, emotions, and reactions of the child in therapy-relevant difficult situations (measured with the video diary function of the therapy app).

Sociodemographic variables (e.g., gender of the patient, age of patients and parents/foster parents, receipt of social welfare assistance by the family/foster family, socioeconomic status of the family/foster family) will be assessed via parent/caregiver interview at T1.

The chronicity of AD symptoms of the child will be assessed with the DADYS-PI (see above) at T1.

The severity of AD symptoms will be assessed at T2 in blinded clinical rating as well as patient, parent/caregiver and teacher rating using the DADYS-PI/questionnaires (see above).

The severity of comorbid symptoms of the child will be assessed at T2 using the SBB-ADHS/−SSV, FBB-ADHS/−SSV, CBCL/6-18R, and TRF/6-18R (see above). Additionally, at T1, comorbid symptoms will be assessed in clinical rating, based on parent/caregiver interview before treatment using a structured screening interview which is part of the comprehensive Structured Interview for Children and Adolescents according to ICD-10 and DSM-5 from the DISYPS-III (DISYPS-III-ILF) [Görtz-Dorten & Döpfner, in preparation]. ILF-SCREEN is a semi-structured interview that is used to screen for core symptoms of ADHD, ODD, anxiety disorders, depression, autism and problems with contact behavior in children. For use in ADOPT Treatment, items concerning developmental and excretion disorders will be excluded. If ILF-SCREEN provides evidence of comorbid symptoms, additional scales from the DISYPS-III clinical interviews, corresponding to these symptoms, will be employed (ILF-External, ILF-Internal, ILF-Kontakt).

AD symptoms of the parent/foster parent participating in the treatment of the child will be measured at T1 via self-rating using the Aggression/Hostility subscale from the German Brief Symptom Checklist (BSCL) [88]. The scale consists of five items that are rated by the participating parent.

Psychopathology of parents and foster parents will be assessed at T1 with the 9-item self-report short version of the Symptom Checklist (SCL-K-9) [89].

Positive and negative parenting practices will be assessed at T1-T4 via rating of the participating parent/caregiver using the German Questionnaire for Positive and Negative Parenting (FPNE) [90], which measures positive parenting (e. g. reinforcing and supportive) and negative parenting (e. g. harsh and inconsistent discipline).

Early childhood neglect and traumatic events of the patient will be assessed at T1 via parent/caregiver questionnaire using a modified version of the Symptom Checklist for Attachment Disorders from the DISYPS-III [80] and the Symptom Checklist for Posttraumatic Disorders from the DISYPS-III [80].

Family climate will be assessed at T1 in parent/foster parent rating using the Family Climate Scale [91].

Social support will be assessed at T1 in parent/caregiver rating using the Social Support Scale (SSS) – Short Version [92].

Personal resources of the child will be assessed with three items from the Self-Efficacy Scale [93], one item from the Bern Well-Being Questionnaire [94] and one item from the Sense of Coherence Scale [95]. The items were modified for the assessment in parent/caregiver rating.

The profile of usage will be analyzed to identify the frequency and duration of usage and the selection of ADOPT Online modules.

Implementation of treatment modules, treatment adherence and treatment fidelity will be rated by the therapists in the THOPAS group after each session of treatment.

For the assessment of the child’s current mood using the momentary assessment function of the therapy app, the child will be asked to indicate how he/she is feeling three times per day over three five-day periods (at the beginning, in the middle and at the end of treatment). Different basic emotions will be presented and the child can choose between the emotions and indicate how strong the emotion/s is/are perceived to be at the specific moment. Additionally, four items from the patient-rated DADYS questionnaire (see above) will be included in the momentary assessment function.

For the assessment of behavior, thoughts, emotions, and reactions of the child in therapy-relevant difficult situations using the video diary function of the therapy app, the child will be asked to “tell” the app about a specific situation and to record it using the video function. The child will then be asked to indicate the specific behavior, thoughts, emotions and reactions that occurred in the situation.

#### Mediators of change

Potential mediators will be assessed at T1-T4 and at the intermediate measurement T1b/T2b: (1) parent/caregiver-reported positive and negative parenting practices (measured with the FPNE, see above), and (2) use of strategies to regulate affect and to reduce anger as reported by the patient and the parent/caregiver. Additionally, the child’s AD symptoms will also be assessed at the intermediate measurements using patient- and parent/caregiver-rated DADYS questionnaires (see above) to determine whether changes in the potential mediators preceded changes in outcome.

For the assessment of strategies to regulate affect and reduce anger, as reported by the patient and the parent/caregiver, we adapted the German questionnaire for the assessment of emotion regulation (FEEL-KJ) [96]. The questionnaire for patient rating was slightly modified and enhanced. The items were then reworded for parent/caregiver rating.

Strategies to regulate emotions of the child by the parent/caregiver will be assessed in parent/caregiver report with a modified version of the Coping with Children’s Negative Emotions Scale (CCNES; German Version VEEB) [97].

### Sample size

In total, *n* = 597 children will be recruited for ADOPT Online. The community sample will be drawn by ADOPT Epidemiology. A screening tool for AD in children and clinician’s rating in the outpatient sample will then be used to identify the AD and the No AD sample. At T1, inclusion and exclusion criteria will be evaluated for the AD sample, resulting in an estimated sample size of *n* = 497 children, who will be randomized with an allocation rate of 5 (ADOPT Online; *n* = 414) to 1 (TAU; *n* = 83).

In ADOPT Treatment, *n* = 232 children need to be recruited and randomized with an allocation rate of 1 (THOPAS; *n* = 116) to 1 (TAU; *n* = 116). T3 assessment needs to include *n* = 198 patients from the treatment and control group (232 patients minus 34 lost at T3, i.e., 20%). At T4, a total of *n* = 158 patients will be assessed for further follow-up (assuming a further dropout of 20% from T3).

As the study designs of ADOPT Online and ADOPT Treatment include a stepped care process, the sample size is defined by the expected effect sizes of the ADOPT Treatment step. The effect sizes found in the reported meta-analyses of trials on the effects of mostly standardized treatments in a group format (mostly compared with waiting list control) are in the range of *d* = 0.3 to 0.6. The effect sizes of personalized individual treatments tend to be elevated, in the range of 0.6 to 0.8 [51, 98]. The planned trial will be conducted with online treatment-refractory AD and may therefore comprise a more severe group of patients, who may be harder to treat. Thus, a moderate effect size of *d* = 0.5 is expected. The two-sample *t*-test with an allocation ratio of 1:1 (THOPAS: TAU) requires 64 patients per group in order to attain a power of 80% at a two-sided significance level of 5%. A similar therapist effect (due to clustering) as in [99] yields a design effect of 1.4 (intraclass correlation 0.1, five patients per therapist on average); thus, *n* = 179 [128*1.4] patients are required [100]. Further accounting for heterogeneous cluster sizes (+ 10%) and attrition (+ 15%) yields approximately *n* = 232 patients to be randomized (*n* = 116 to THOPAS, *n* = 116 to TAU). Note that power is further increased by taking a baseline-adjusted analysis of covariance (ANCOVA)/mixed model for repeated measures (MMRM) approach for statistical analysis. We expect a male:female ratio of 7:3 [51]. Thus, the sample size of ADOPT Treatment is set at *n* = 232, with *n* = 116 in the treatment arm and *n* = 116 in the control group.

Meta-analyses of self-help PMT report effect sizes between 0.61 and 1.01 [25, 27], with smaller effects for self-help interventions with no therapist contact. It remains unclear whether symptom severity serves as a moderator favoring severely affected children in PMT [22]. Breitenstein [27] reported mean completion rates of about 78% in their meta-analysis on online PMT. In studies on self-help PMT, dropout ranged between 5 and 30% [28, 56]. On the other hand, for highly burdened parents, like those in our AD sample, online interventions might be harder to successfully complete. Given this, and the fact that online PMT without therapist contact is already less effective than telephone-assisted self-help interventions, a small effect size such as 0.378 should be expected for ADOPT Online. Thus, the two-sample t-test with an allocation ratio of 5:1 (ADOPT Online: TAU) requires 331 and 66 patients, respectively, to attain a power of 80% at a two-sided significance level of 5%. Further accounting for 20% attrition yields about *n* = 497 [≈ 397/0.8] patients to be randomized (*n* = 414 to ADOPT Online, *n* = 83 to TAU). Note that power is further increased by taking a baseline-adjusted ANCOVA/MMRM approach for statistical analysis. We expect a male:female ratio of 7:3 [51]. Missing data and non-compliance will be dealt with by performing intention-to-treat (ITT) analysis.

In ADOPT Institution, a screening tool for AD in children will then identify the AD (*n* = 166) and the No AD (*n* = 80) sample in a total of *n* = 750 children in OHC. According to the cut-off developed in ADOPT Epidemiology, we expect that at least 25% of the screening sample will be identified as screening-positive. Since the institutions have already agreed to take part in the project, we expect that most of the children screened positive will participate in the intervention. A total of *n* = 166 children need to be recruited and randomized with an allocation rate of 1 (THOPAS) to 1 (TAU) (83 to the experimental treatment, 83 to TAU). T2 assessment needs to be conducted with *n* = 140 patients from the treatment and control group (166 patients minus 26 lost at T2, i.e., 20%) and 64 from the No AD group. At T4, a total of 169 patients will be assessed for further follow-up (assuming a further dropout of 20% from T2).

The planned trial will be conducted with children in OHC, who presumably have a higher comorbidity load and may therefore constitute a more severe group of patients, who may be harder to treat. Thus, a moderate effect size of *d* = 0.6 is expected. The two-sample t-test with an allocation ratio of 1:1 (THOPAS:TAU) requires 45 patients per group to attain a power of 80% at a two-sided significance level of 5%. A similar therapist effect (due to clustering) as in [99] yields a design effect of 1.4 (intraclass correlation 0.1, five patients per therapist on average); thus, *n* = 126 [≈(45 + 45)*1.4] patients are required. Further accounting for heterogeneous cluster sizes (+ 10%) and attrition (+ 15%) yields about *n* = 166 [≈126/0.9/0.85] patients to be randomized (*n* = 83 to THOPAS, *n* = 83 to TAU). Based on previous experience, we expect that 664 patients need to be screened for eligibility (i.e. only 25% are eligible). Note that power is further increased by taking a baseline-adjusted ANCOVA/ MMRM approach for statistical analysis. We expect a male:female ratio of 7:3 [39].

### Data management and confidentiality

ADOPT Coordination, in cooperation with the Clinical Trials Center Cologne (CTCC), will provide electronic questionnaires within the framework of a remote data entry system (REDCap) [101] and will administer the database. REDCap is a proprietary remote data entry system which was developed by Vanderbilt University, Nashville, TE, USA. An instance of REDCap is hosted and maintained by the CTCC. Each investigator/data entry personnel will receive separate access information for the use of the REDCap system. An audit trail within REDCap provides a data history of which data were entered, changed or deleted, when and by whom. The data will be reviewed for completeness, consistency and plausibility. Data corrections will be entered directly into REDCap by the responsible investigator or a designated person.

Details of the data management (procedures, responsibilities, timelines, data validation and data corrections) will be described in a data management manual (DMM) prior to trial start. The DMM is a working document that is adapted during the clinical trial and contains a record of all data management processes carried out.

Each patient will receive a consecutive patient identification number (ID) after screening. The ID will be entered in the centralized database (REDCap) for the registration of the patient.

For ADOPT Institution, the ID will be assigned by the person who completes the screening questionnaire (i.e., the caregiver) with instructions provided by ADOPT Institution. Thus, screening can be performed without disclosure of identifying information of the child in the study center. After identification of a screening-positive case and after informed consent of the guardians has been obtained, the child and caregiver will be invited for T1 assessment and further participation in ADOPT Institution.

ADOPT Coordination, in cooperation with the CTCC and the PIs, will process data through personnel who are specifically trained for the study, and who will then work in accordance with the standard operating procedures (SOPs) of the study centers. Legal regulations for data protection will be fulfilled.

### Statistical analysis

#### Patient demographics/other baseline characteristics

Demographic and other baseline data (including disease characteristics) will be obtained at T1 and will be summarized descriptively using all documented patients.

Continuous data will be summarized by arithmetic mean, standard deviation, minimum, 25% quantile, median, 75% quantile, maximum, and the number of complete and missing observations. If appropriate, continuous variables can also be presented in categories.

Categorical data will be summarized by the total number of patients in each category and the number of missing values. Relative frequencies will be displayed as valid % (number of patients divided by the number of patients with non-missing values).

#### Analysis of primary endpoint

The primary (full) analysis set is derived from the ITT principle (all patients randomized with a valid baseline assessment: ADOPT Online/ADOPT Institution: T1/ADOPT Treatment: T2 and at least one follow-up measurement). The primary outcome measures “change in AD symptoms from T1 to 13 weeks post-randomization (T2)” (ADOPT Online), “change in AD symptoms from T2 to 32 weeks post-randomization (T3)” (ADOPT Treatment) and “change in AD symptoms from T1 to 32 weeks post-randomization (T2)” (ADOPT Institution) will be evaluated by an ANCOVA, with fixed effects baseline, study center, gender and treatment arm and corresponding marginal means and contrast tests (type II sums of squares). Interactions of study center and gender with treatment arm will be explored. Data from study centers with low recruitment (i.e., < 10 patients) will be pooled. The potential clustering of observations of participants by therapist or center will be investigated using multilevel modelling. Multiple imputation approaches will be taken to assess the robustness of the results. Specifically, missing values will be separately imputed by type assuming mixtures of missingness-not-at-random patterns [102]. Imputation data sets will be post-processed by multiplication with factors and addition of offsets (tipping point analysis) [103]. Proxy measures will be taken into account to ameliorate the effects of attrition.

#### Analysis of secondary endpoints

Secondary outcomes (i.e., further time points and measures) will be analyzed along the same lines, possibly using MMRM (heterogeneous first-order autoregressive-structured covariance matrix over time) or generalized estimating equation approaches with corresponding marginal means and contrast tests (“multilevel modelling”). Time-to-dropout distributions will be summarized by the Kaplan-Meier method and compared using the (stratified) log-rank test. Adverse events will be aggregated by type, seriousness, intensity and relatedness. All efficacy and safety variables will be summarized by time point and treatment arm (mean, standard deviation, percentiles (i.e., minimum, 25th, 50th, 75th, and maximum), count, percentage). Subgroup analyses will be conducted according to recruitment process, study center and gender (expected male to female ratio of 7:3, thus, meaningful results are expected for boys and girls). Moreover, moderation, mediation and conditional process modelling [104] will be conducted based on regression and structural equations (interaction, simple slope analysis; direct/indirect effects, kappa square). All of the details, particularly regarding how to deal with missing data and attrition, will be documented in a statistical analysis plan.

#### Missing values

It should be emphasized that as few patients as possible should discontinue treatment and that all patients should be followed up and also documented after discontinuation of the treatment in order to record data required according to the ITT principle. To assess the impact of up to 20% attrition, multiple imputation approaches will be taken, accounting for proxy measures and assuming specific missingness-not-at-random patterns. The details will be documented in a statistical analysis plan. Analysis of subjects essentially observed and treated per protocol is supportive.

#### Safety

Safety analyses will be performed in the safety population. Patients in the safety population will be analyzed as belonging to the treatment arm defined by treatment received (ADOPT Online or TAU; ADOPT Treatment/ADOPT Institution: THOPAS or TAU). Patients will be included in the respective treatment arm if treatment was started/if they received at least one dose of trial treatment. Patients who refuse participation in ADOPT Online/THOPAS will also be part of the safety population.

### Data monitoring

#### Data monitoring committee

A Data Monitoring Committee (DMC) has been established. The DMC comprises three experts with expertise in conducting clinical trials and specific expertise in biostatistics, psychotherapy, and child and adolescent psychiatry. Prior to the implementation of the trials, a DMC charter will be worked out, describing goals and the work plan of the board. The DMC will assess, on an annual basis, whether the execution of the study is still ethically justified and whether performance is acceptable.

#### Harms

In the framework of the study, the evaluation of tolerability/safety will be restricted to the occurrence of serious adverse events (SAEs). An SAE is defined as an event that: (1) results in the participant’s death, (2) is a suicide attempt; (3) results in hospitalization for non-suicidal self-harm; or (4) results in hospitalization for mental health problems. All serious adverse events will be reported to the independent DMC.

#### Auditing

Monitoring will be performed by the CTCC in cooperation with the ADOPT Coordination subproject. All investigators agree that the monitor is allowed to visit the center before and during the study. The pre-study monitoring visit and all other monitoring visits will be performed by the CTCC in accordance with the trial protocol, the established quality management system (SOPs), and guidelines of the International Conference on Harmonization of Good Clinical Practice (ICH-GCP). The results of the pre-study visits will be documented and reported back to the funding agency. The monitoring strategy will be specified within a study-specific monitor manual.

### Stopping rules

#### Stopping rules for an individual patient

One (or more) of the following circumstances will result in an early study termination of single subjects (these trial subjects will be rated as dropouts): (i) withdrawal of informed consent of all parents/guardians; (ii) withdrawal of assent of the patient; (iii) unwillingness to further participate in the trial; (iv) need for inpatient treatment or other reasons affecting the patient’s well-being in the case of continued trial participation; (v) need for a different kind of treatment for health reasons according to the judgment of the attending physician.

#### Global stopping rules

A termination of the entire consortium will be executed if both ADOPT Epidemiology and ADOPT Institution fail to reach 50% of the planned sample size, despite additional recruitment strategies. The decision will be made by all principal investigators as well as the principal coordinator.

## Discussion

The ADOPT study will inform about the benefits of online self-help for parents and intensive personalized modular treatments compared to standard approaches. These effects will be related to AD symptoms, comorbid symptoms, functional impairment, and psychological well-being. The study will also inform about putative predictors, moderators and mediators of intervention effects on AD. The modular approach will have a strong impact on clinical practice, as it will help clinicians to adapt their treatment to the needs of the individual patient. For children living in OHC, especially effects on functional impairment and quality of life, but also effects on multiple placements or early discontinuation of youth welfare interventions, will be of high relevance for the individual child, but also of economic relevance for the health care and youth welfare system.

The results will have a large impact on clinical practice, as they will (1) improve assessment of the clinical condition (also in a high-risk population of children living in OHC), (2) inform about the relation of AD and clinical diagnoses of co-existing disorders and about risk and protective factors, (3) introduce personalized modular psychological transdiagnostic treatment and increase treatment effects. Since AD is an important risk factor for the development of severe mental disorders, the results should affect the health status of the pediatric target population in later periods of life. Therefore, the results of the proposed project will be highly relevant for the assessment and treatment of children with AD and comorbid conditions.

The results of the ADOPT study will inform future guidelines on the treatment of children with AD and comorbid conditions and will help to improve guidelines and to develop usable, potentially more cost-effective, individualized modular treatment in children with AD in the mental health care system and youth welfare system.

## Additional files

Additional file 1: Trial registration ADOPT Online. (PDF 225 kb)

Additional file 2: Trial registration ADOPT Treatment. (PDF 343 kb)

Additional file 3: Trial registration ADOPT Institution. (PDF 334 kb)

Additional file 4: Model written informed consent. (PDF 531 kb)

## Data Availability

After publication of the results, the final datasets can be obtained from the principal investigators of each trial (ADOPT Online: Charlotte Hanisch; ADOPT Treatment: Anja Görtz-Dörten; ADOPT Institution: Jörg M. Fegert or Michael Kölch) on reasonable request.

## Abbreviations

AD: Affective dysregulation
ADHD: Attention-deficit/hyperactivity disorder
ADOPT Online: Online Parent Self-Help of Affective Dysregulation and coexisting disorders
ANCOVA: Analysis of covariance
ARI: Affective Reactivity Index
BSCL: Brief Symptom Checklist
CBCL/6-18R: Child behavior checklist (parent-rated)
CBCL-DP: Child behavior checklist dysregulation profile
CBT: Cognitive behavior therapy
CD: Conduct disorder
CCNES: Coping with Children’s Negative Emotions Scale
CSQ: Client Satisfaction Questionnaire
CTCC: Clinical Trials Center Cologne
DADYS: Diagnostikum für Affektive Dysregulation bei Kindern (Diagnostic tool for affective dysregulation in children)
DADYS-CI: DADYS child interview
DADYS-PI: DADYS parent interview
DADYS-Screen: DADYS screening questionnaire
DISYPS-III: Diagnostic System for Mental Disorders in Children and Adolescents
DMC: Data Monitoring Committee
DMM: Data management manual
DRKS: Deutsches Register Klinischer Studien (German clinical trials register)
DSM-5: Diagnostic and statistical manual of mental disorders of the American Psychiatric Association, 5th edition
ERC: Emotion Regulation Checklist
FBB-ADHS: Fremdbeurteilungsbogen ADHS (rating scale for ADHD)
FBB-BIST: Fremdbeurteilungsbogen für Bindungs- und Beziehungsstörungen (rating scale for attachment and relationship disorders)
FBB-SCREEN: Fremdbeurteilungsbogen screening (rating scale for screening)
FBB-SSV: Fremdbeurteilungsbogen SSV (rating scale for oppositional defiant disorder and conduct disorder)
FBB-TBS: Fremdbeurteilungsbogen für Trauma- und Belastungsbezogene Störungen (rating scale for trauma- and stress-related disorders)
FEEL-KJ: Fragebogen zur Erhebung der Emotionsregulation bei Kindern und Jugendlichen (questionnaire for the assessment of emotion regulation)
FPNE: Fragebogen zum positiven und negativen Erziehungsverhalten (questionnaire on positive and negative parenting behavior)
ICD-10: International statistical classification of diseases and related health problems, 10th revision
ICD-11: International statistical classification of diseases and related health problems, 11th revision
ICH-GCP: International Conference on Harmonization of Good Clinical Practice
ID: Identification number
ILF-External: Structured interview for externalizing symptoms
ILF-Internal: Structured interview for internalizing symptoms
ILF-Kontakt: Structured interview for contact-related symptoms
ILF-SCREEN: Structured screening interview
IMSB: Institute of Medical Statistics and Computational Biology
ITT: Intention-to-treat
MATCH-ADTC: Modular approach to therapy for children with anxiety, depression, trauma, or conduct problems
MMRM: Mixed model for repeated measures
No AD: Children without affective dysregulation
ODD: Oppositional defiant disorder
OHC: Out-of-home care
PMT: Parent management training
PROMIS: Patient-Reported Outcomes Measurement Information System
RCI: Reliable change index
RCT: Randomized controlled trial
RDoC: National Institute of Mental Health Research Domain Criteria
REDCap: Remote data entry system provided by the Clinical Trials Center Cologne
SAE: Serious adverse event
SBB-ADHS: Selbstbeurteilungsbogen ADHS (self-rating scale for ADHD)
SBB-SSV: Selbstbeurteilungsbogen SSV (self-rating scale for oppositional defiant disorder and conduct disorder)
SCEP: School-based Coaching for Teachers of Children with Disruptive Behavior Problems
SCL-K-9: 9-item self-report short version of the Symptom Checklist
ScouT: Social Computer-assisted Training for Children with Aggressive Behavior Problems
SOP: Standard operating procedure
SSS: Social Support Scale – short version
TAU: Treatment as usual
THAV: Treatment Program for Children with Aggressive Behavior Problems
THOP: Therapieprogramm für Kinder mit hyperkinetischem und oppositionellem Problemverhalten (therapy program for the treatment of children with hyperkinetic and oppositional problem behavior)
THOPAS: Therapieprogramm zur Optimierung Affektiver Steuerung bei Kindern (Therapy for Optimizing Affective Regulation in Children)
TRF/6-18R: Teacher’s report form
VEEB: Fragebogen zum Verhalten von Eltern bei emotionalen Belastungen ihrer Kinder (Questionnaire on coping of parents with children’s emotional distress)
