# Supplementary material for: Affective dysregulation in childhood - optimizing prevention and treatment: protocol of three randomized controlled trials in the ADOPT study
Source: BMC Psychiatry. 2019 Sep 2;19:264. doi: 10.1186/s12888-019-2239-8 (PMC6720991; doi:10.1186/s12888-019-2239-8)
Supplement: Supplementary file 1 — Trial registration ADOPT Online. (PDF 225 kb) [file 12888_2019_2239_MOESM1_ESM.pdf]

Additional file 1: Trial registration ADOPT Online

DRKS-ID: DRKS00014963
Trial Description

Title

Efficacy of Internet Based Parent Management Training in the Treatment of Affective Dysregulation and Coexisting Conditions in Children

Trial Acronym

ADOPT Online

URL of the Trial

http://www.adopt-studie.de

Brief Summary in Lay Language

When children suffer from problems in dealing with negative emotions like anger or sadness we call this Affective Dysregulation. Those children show bursts of anger, persistent irritability, bad temper and sudden mood changes. The aim of this study is to develop an online parent training in which parents learn strategies to solve daily problems with their child and to support their child in dealing with negative emotions. Additionally, we will investigate whether the training helps.

Brief Summary in Scientific Language

This subproject is embedded into a study consortium that develops and evaluates a stepped intervention for children with Affective Dysregulation at five sites in Germany. In this subproject called ADOPT online, the efficacy of an Online Parent Self-Help of Affective Dysregulation and coexisting disorders (OnPaSH-AD) is to be investigated compared to treatment as usual. The term Affective Dysregulation describes a transdiagnostic dimension and characterizes an excessive reactivity to negative emotional stimuli with an affective (anger) and a behavioral component (aggression). An evaluated online training for parents of children with ADHD will be supplemented with evidence-based interventions for the improvement of emotion regulation. In a randomized control trial the efficacy will be assessed over a reduction of symptoms of affective dysregulation, comorbid conditions, functional impairment and psychosocial wellbeing.
Amendment: Reduction of sample size after renewed power calculation (vote in favour on 22.02.2019)

Organizational Data

- DRKS-ID: DRKS00014963
- Date of Registration in DRKS: 2018/06/27
- Investigator Sponsored/Initiated Trial (IST/IIT): yes
- Ethics Approval/Approval of the Ethics Committee: Approved
- (leading) Ethics Committee No.: 18-033, Ethik-Kommission der Medizinischen Fakultät der Universität zu Köln

Health Condition or Problem studied

ICD10: F34.8 Other persistent mood [affective] disorders
Free text: mood dysregulation as transdiagnostic dimension

Interventions/Observational Groups

- Arm 1: Intervention group: online parent training at least one hour weekly for 12 weeks
- Arm 2: Treatment as usual: Parents of this group are advised to search for a psychosocial support if wanted (e.g. education counselling, outpatient psychotherapy) or to wait. After a 12 weeks waiting phase they gain access to the online parent training.

Characteristics

- Study Type: Interventional
- Allocation: Randomized controlled trial
- Blinding: Blinded
- Who is blinded: assessor
- Control: Other
- Purpose: Prevention
- Assignment: Parallel

Primary Outcome

Blinded clinician-rated AD (=Affective Dysregulation) symptom score of the child, assessed with a newly developed outcome measure for AD (DADYS interview) based on patient and parent interview at T1-T4

Secondary Outcome

(1) psychosocial impairment of the child due to AD symptoms based on patient and parent interview (DADYS interview/DADYS questionnaire)
(2) patient- and parent-rated AD symptoms of the child (DADYS questionnaire)
(3) patient- and parent-rated symptoms of ADHD and ODD/CD (SBB-ADHS/-SSV, FBB-ADHS/-SSV)
(4) other comorbid conditions (e.g., anxiety, depression) assessed by parent-ratings (CBCL 6-18R)
(5) psychological well-being in patient- and parent-rating (KIDSCREEN-27)
(6) parental satisfaction with the treatment (self-developed questionnaire)
(1)-(5) will be collected at T1-T4; (6) will be collected at T2

Countries of Recruitment

DE: Germany

Locations of Recruitment

- University Medical Center: Uniklinik Köln, Klinik und Poliklinik für Psychiatrie, Psychosomatik und Psychotherapie des Kindes- und Jugendalters, Ausbildungsinstitut für Kinder- und Jugendlichenpsychotherapie (AKiP), Köln
- Other: Universität zu Köln, Humanwissenschaftliche Fakultät, Ki-Ju-Ambulanz, Köln
- University Medical Center: Universitätsklinik Hamburg-Eppendorf, Klinik für Kinder- und Jugendpsychiatrie, -psychotherapie und -psychosomatik, Forschungsabteilung Child Public Health, Hamburg
- University Medical Center: Universitätsklinikum Dresden, Klinik und Poliklinik für Kinder- und Jugendpsychiatrie und -psychotherapie, Dresden
- University Medical Center: Zentralinstitut für seelische Gesundheit, Klinik für Psychiatrie und Psychotherapie des Kindes- und Jugendalters, Mannheim
- Medical Center: Ruppiner Kliniken, Klinik für Kinder- und Jugendpsychtrie , Neuruppin
- University Medical Center: Universitätsklinikum Ulm, Klinik für Kinder- und Jugendpsychiatrie/Psychotherapie, Psychiatrische Institutsambulanz für Kinder und Jugendliche (KJPIA), Ausbildungszentrum für Verhaltenstherapie (AZVT), Ulm

Recruitment

- Planned/Actual: Actual
- (Anticipated or Actual) Date of First Enrollment: 2018/07/09
- Target Sample Size: 497
- Monocenter/Multicenter trial: Multicenter trial
- National/International: National

Inclusion Criteria

- Gender: Both, male and female
- Minimum Age: 107 Months
- Maximum Age: 155 Months

Additional Inclusion Criteria

- Age 8;0 to 12;11 yrs.
- Resident with at least one natural or adoptive parent
- Clinician-rated Outcome Measure for AD (DADYS interview, DADYS questionnaire) > cut-off. The cut-off will be determined by a clinical overall rating of AD at the end of the structured clinical parent interview.
- Willingness and ability of parents to participate in the online intervention (existence of an informed consent of the guardians and an assent of the child)
- Sufficient knowledge of German language

Exclusion Criteria

- Intelligence <80 (visit to a special school for mental development or to a regular school with funding priority mental development)
- Resident without natural or adoptive parents (e.g. foster parents, grandparents)
- mental disorder other than Coexisting Conditions (CoCo) is primary disorder and main cause of AD (e.g. autism spectrum disorder)
- current or planned intensive behavioral therapy on a weekly/biweekly basis

Addresses

Primary Sponsor

Universität zu Köln
Humanwissenschaftliche Fakultät
Psychologie und Psychotherapie in Heilpädagogik und Rehabilitation
Ms. Prof. Dr. Charlotte Hanisch
Klosterstraße 79b
50931 Köln
Germany
Telephone: 02214705520
Fax: 02214705576
E-mail: charlotte.hanisch@uni-koeln.de
URL: https://www.hf.uni-koeln.de/621

Contact for Scientific Queries

Universität zu Köln
Humanwissenschaftliche Fakultät
Psychologie und Psychotherapie in Heilpädagogik und Rehabilitation
Ms. Anne Ritschel
Klosterstraße 79b
50931 Köln
Germany
Telephone: 022147076181
Fax: 02214705576
E-mail: anne.ritschel@uni-koeln.de
URL: https://www.hf.uni-koeln.de/621

Contact for Public Queries

Universität zu Köln
Humanwissenschaftliche Fakultät
Psychologie und Psychotherapie in Heilpädagogik und Rehabilitation
Ms. Anne Ritschel
Klosterstraße 79b
50931 Köln
Germany
Telephone: 022147076181
Fax: 02214705576
E-mail: adopt-studie@uni-koeln.de
URL: https://www.adopt-studie.de/

Sources of Monetary or Material Support

Public funding institutions financed by tax money/Government funding body (German Research Foundation (DFG), Federal Ministry of Education and Research (BMBF), etc.)
BMBF
53170 Bonn
Germany

Status

Recruitment Status: Recruiting ongoing

Trial Publications, Results and other Documents

trial protocol (mandatory for transfer to Studybox): Studienprotokoll ADOPT Online
tudienmitarbeiters/in
