# Supplementary material for: Affective dysregulation in childhood - optimizing prevention and treatment: protocol of three randomized controlled trials in the ADOPT study
Source: BMC Psychiatry. 2019 Sep 2;19:264. doi: 10.1186/s12888-019-2239-8 (PMC6720991; doi:10.1186/s12888-019-2239-8)
Supplement: Supplementary file 2 — Trial registration ADOPT Treatment. (PDF 343 kb) [file 12888_2019_2239_MOESM2_ESM.pdf]

Additional file 2: Trial registration ADOPT Treatment

DRKS-ID: DRKS00013317

Trial Description

Title

Efficacy of a Personalized Modular Outpatient Treatment of Affective Dysregulation and Coexisting Conditions in Children

Trial Acronym

ADOPT Treatment

URL of the Trial

http://www.adopt-studie.de

Brief Summary in Lay Language

In the last years, researchers recognized a growing amount of children and adolescents that have problems to regulate or to reduce negative emotions like anger or sadness. This is called affective dysregulation. ADOPT Treatment aims at investigating the efficacy of a new treatment approach for children with affective dysregulation. The study will include children at the age of 8 to 12 years, whose parents already took part in a parent management training and who show still signs of affective dysregulation. The children receive either an individualized psychotherapy or are treated as usual. About 250 families will take part in the study at five study sites in Germany. We will investigate if the psychotherapy is effective in reducing the affective dysregulation and if other, associated problems, will decrease too. Further, we will examine if certain children or families have a better outcome than others and through which mechanisms the treatment works.

Brief Summary in Scientific Language

The term Affective Dysregulation (AD) describes a transdiagnostic dimension and characterizes an excessive reactivity to negative emotional stimuli with an affective (anger) and a behavioral component (aggression). AD is a criterion for several DSM-5/ICD-10 diagnoses. Most traditional evidence-based cognitive behavioral therapies focus on one disorder (or a small cluster of related disorders), making it difficult for clinicians to address heterogeneous caseloads and client comorbidities. Therefore, a more flexible modular approach to therapy for children is needed in order to work across a range of these disorders associated with clinical problems. The study has several aims: (1) To determine the efficacy of a personalized modular outpatient treatment of AD and coexisting disorders in comparison to treatment as usual in children aged 8;0 to 12;11 yrs. with substantial residual symptoms of AD or coexisting conditions after receiving an internet based parent management training; (2) to evaluate the feasibility of the implementation in routine care; (3) to evaluate predictors, moderators, mediators and stability of treatment outcome. Primary efficacy measure will be the change in blinded clinician-rated AD symptom score of the child based on parent interview.

Organizational Data

- DRKS-ID: DRKS00013317
- Date of Registration in DRKS: 2018/09/27
- Investigator Sponsored/Initiated Trial (IST/IIT): yes
- Ethics Approval/Approval of the Ethics Committee: Approved
- (leading) Ethics Committee No.: 18-040, Ethik-Kommission der Medizinischen Fakultät der Universität zu Köln

Health Condition or Problem studied

Free text: Affective dysregulation
ICD10: F90.0 Disturbance of activity and attention
ICD10: F91.3 Oppositional defiant disorder
Other: DSM 5: 296.99 (Disruptive mood dysregulation disorder)
ICD10: F93.8 Other childhood emotional disorders

Interventions/Observational Groups

- Arm 1: Personalized modular outpatient treatment of affective dysregulation and coexisting disorders with THOPAS: THOPAS consists of 10 CBT (Cognitive Behavior Therapy) modules that include child-based intervention, parent training interventions and teacher coaching. The child-centered interventions are based on evidence-based German treatment programs – the Treatment Program for Children with Aggressive Behavior Problems (THAV; Görtz-Dorten & Döpfner, 2010), and the Social Computer-assisted Training for Children with Aggressive Behavior Problems (ScouT; Görtz-Dorten & Döpfner, 2016) – which are adapted from evidence-based international treatment manuals, especially the Coping

Power Program (Lochman, Wells, & Lenhart, 2008), the Cognitive-Behavioral Therapy for Anger and Aggression in Children (Sukhodolsky & Scahill, 2012), the Organizational Skills Training (Gallagher, Abikoff, & Spira, 2014), and the Modular Approach to Therapy for Children with Anxiety, Depression, Trauma, or Conduct Problems (MATCH-ADTC; Chorpita & Weisz, 2009). The child-centered interventions also include guidance for parents/teachers to support the behavior change of the children at home/school. The parent training interventions are based on the evidence-based Treatment Program for Hyperkinetic and Oppositional Problem Behavior (THOP; Döpfner, Schürmann, & Frölich, 2013) which is adapted from evidence-based international treatment manuals, especially Barkleys Defiant Children (Barkley, 2013), and Helping the Non-compliant Child (McMahon & Forehand, 2003). The teacher coaching is also based on the THOP-Program and a newly developed German School-based Coaching for Teachers of Children with Disruptive Behavior Problems (SCEP; C. Hanisch, Richard, Eichelberger, Greimel, & Döpfner, 2017) which is adapted from evidence-based international treatment manuals, especially DuPaul and Stoner (2014).

- Arm 2: The control intervention includes a treatment as usual (TAU) with a duration of intervention per patient of 32 weeks. TAU as control condition informs about the additional benefit of THOPAS compared to usual care. Within TAU, all psychosocial, psychotherapeutic and pharmacological interventions will be documented.

Characteristics

- Study Type: Interventional
- Allocation: Randomized controlled trial
- Blinding: Blinded
- Who is blinded: assessor
- Control: Active control (effective treament of control group)
- Purpose: Treatment
- Assignment: Parallel

Primary Outcome

Primary efficacy outcome is the blinded clinician-rated affective dysregulation symptom score of the child, assessed with a newly developed outcome measure for AD (DADYS interview) based on parent interview. DADYS will be developed by another subproject of the ADOPT consortium (ADOPT Epidemiology, in cooperation with ADOPT Treatment) before the start of the trial. Affective dysregulation of the child will be measured before and after treatment and after a 8-months follow-up.

Secondary Outcome

The secondary endpoints are also summary scores/scale scores of the respective items. The secondary outcomes listed below will be evaluated at T2-T4, unless otherwise noted.

Secondary outcomes are (1) psychosocial impairment of the child due to AD symptoms in blinded clinical rating as well as patient-, parent-, and teacher-rating (DADYS interview/DADYS questionnaire), (2) patient-, parent-, teacher- and clinician-rated AD symptoms of the child (DADYS questionnaire, DADYS interview), (3) patient-, parent- and teacher-rated symptoms of ADHD and ODD/CD (SBB-ADHS/-SSV, FBB-ADHS/-SSV; FBB-Screen), (4) other comorbid conditions (e.g., anxiety, depression) assessed by parent-ratings and teacher-ratings (CBCL 6-18R, TRF 6-18R, FBB-BIST [only parent-rating], FBB-TBS [only parent-rating]), (5) psychological well-being in patient- and parent-rating (KIDSCREEN-10-Index, KIDSCREEN-27), and (6) satisfaction with the treatment.

As predictors/moderators for treatment outcome on AD symptoms and impairment the following variables will be analyzed: (1) gender, (2) age of patients and parents, (3) chronicity of AD-Symptoms, (4) severity of AD symptoms, (5) severity of comorbid symptoms, (6) AD-symptoms and other psychopathology of the participating parent, (7) positive and negative parenting practices, (8) public assistance of the family, (9) socio-economic status of the family, (10) early childhood neglect of the patient, (11) family climate in perception of the child, (12) social support and (13) personal resources of the child. These parameters are assessed at T1 and/or at T2. Additionally, during treatment, (14) implementation of treatment modules will be assessed and analyzed as predictor for treatment outcome in the THOPAS group as well as (15) treatment adherence rated and (16) treatment fidelity, both rated by the therapist.

Potential mediators are assessed at T2, at intermediate measurement T2b and at T3/T4: (1) parent reported positive and negative parenting practices (measured with the FPNE, see above), and (2) use of strategies to regulate affect and to reduce anger as reported by the patient and the parents. Additionally, AD

symptomatology of the child will also be assessed at intermediate measurements with patient- and parent-rated DADYS questionnaires (see above) to determine if changes in the potential mediators preceded changes in outcome.

Countries of Recruitment

DE: Germany

Locations of Recruitment

- University Medical Center: Ausbildungsinstitut für Kinder- und Jugendlichenpsychotherapie (AKiP) an der Klinik und Poliklinik für Psychiatrie, Psychosomatik und Psychotherapie des Kindes- und Jugendalters, Köln
- Other: Humanwissenschaftliche Fakultät, Universität zu Köln, Köln
- University Medical Center: Klinik und Poliklinik für Kinder- und Jugendpsychiatrie und -psychotherapie, Dresden
- University Medical Center: Klinik für Kinder- und Jugendpsychiatrie/Psychotherapie, Ulm
- Medical Center: Klinik für Psychiatrie und Psychotherapie des Kindes- und Jugendalters des Zentralinstitut für seelische Gesundheit (ZI), Mannheim

Recruitment

- Planned/Actual: Actual
- (Anticipated or Actual) Date of First Enrollment: 2018/10/08
- Target Sample Size: 261
- Monocenter/Multicenter trial: Multicenter trial
- National/International: National

Inclusion Criteria

- Gender: Both, male and female
- Minimum Age: 8 Years
- Maximum Age: 12 Years

Additional Inclusion Criteria

Patients will be included if they meet following inclusion criteria:
Age: 8;0 to 12;11 years (= completed 8th year of life to completed 13th year of life) at T1; resident with at least one natural parent/adoptive parent; clinician-rated AD symptoms of the child (DADYS interview) based on parent interview > cut-off at T2; willingness and ability of patient and parents to participate in the intervention.

Exclusion Criteria

Following exclusion criteria will be applied and evaluated at T1:
Intelligence below average in clinical evaluation/patient attends school for intellectual disabilities; child is not resident with at least one natural parent/adoptive parent; mental disorder other than CoCo is primary disorder and main cause of AD (e.g. autism spectrum disorder); current or planned intensive behavioral therapy or behavioral parent management training on a weekly/biweekly basis

Addresses

Primary Sponsor

Universität zu Köln
Albertus-Magnus-Platz
50923 Köln
Germany

Contact for Scientific Queries

Ausbildungsinstitut für Kinder- und Jugendlichenpsychotherapie an der Klinik und Poliklinik für Psychiatrie, Psychosomatik und Psychotherapie des Kindes- und Jugendalters der Uniklinik Köln
Ms. PD Dr. Anja Görtz-Dorten
Pohligstraße 9
50969 Köln
Germany
Telephone: 0221-478 76823
E-mail: anja.goertz-dorten@uk-koeln.de
URL: http://www.akip.de

Contact for Public Queries

Ausbildungsinstitut für Kinder- und Jugendlichenpsychotherapie an der Klinik und Poliklinik für Psychiatrie, Psychosomatik und Psychotherapie des Kindes- und Jugendalters der Uniklinik Köln
Ms. M. Sc. Psych. Anne-Katrin Treier
Pohligstraße 9
50969 Köln
Germany
Telephone: 0221 478-76837
E-mail: adopt@uk-koeln.de
URL: http://www.adopt-studie.de

Sources of Monetary or Material Support

Public funding institutions financed by tax money/Government funding body (German Research Foundation (DFG), Federal Ministry of Education and Research (BMBF), etc.)
Bundesministerium für Bildung und Forschung Dienstsitz Bonn
Heinemannstr. 2
53175 Bonn
Germany
URL: http://www.bmbf.de

Status

Recruitment Status: Recruiting ongoing

Trial Publications, Results and other Documents

trial protocol (mandatory for transfer to Studybox): Studienprotokoll ADOPT Treatment
