# Supplementary material for: Affective dysregulation in childhood - optimizing prevention and treatment: protocol of three randomized controlled trials in the ADOPT study
Source: BMC Psychiatry. 2019 Sep 2;19:264. doi: 10.1186/s12888-019-2239-8 (PMC6720991; doi:10.1186/s12888-019-2239-8)
Supplement: Supplementary file 3 — Trial registration ADOPT Institution. (PDF 334 kb) [file 12888_2019_2239_MOESM3_ESM.pdf]

Additional file 3: Trial registration ADOPT Institution

DRKS-ID: DRKS00014581

Trial Description

Title

Affective Dysregulation in Childhood – Optimizing Prevention and Treatment (ADOPT): Efficacy of a personalized modular outpatient treatment program to treat affective dysregulation and comorbid disorders in out-of-home care children

Trial Acronym

ADOPT Institution

URL of the Trial

http://www.adopt-studie.de

Brief Summary in Lay Language

ADOPT-Institution is part of a joint research project (ADOPT), aiming at optimizing prevention and treatment of affective dysregulation in children (i.e. reacting overly angry or aggressive in response to provocations). In order to ameliorate prevention approaches, new assessment tools will be developed and evaluated, the frequency of affective dysregulation symptoms will be investigated, and risk and protective factors will be analyzed (sub-project ADOPT-epidemiology). Likewise, an online self-help training program for parents will be developed and evaluated (sub-project ADOPT-online), in which e.g. positive interaction methods will be teached. As there is currently no special treatment program for children with affective dysregulation, the development and evaluation of a modularized psychotherapy program will be subject of two other sub-projects (ADOPT-treatment, ADOPT-institution). While the evaluation sample of ADOPT-treatment will be children, who still live in their family homes, ADOPT-institution will investigate the effects the treatment has on children in out-of-home care. According to the higher amount of adverse childhood experiences in this population, those children are exposed to higher risks in developing symptoms of affective dysregulation.

Brief Summary in Scientific Language

ADOPT-Institution is part of a multi-center joint research project (ADOPT), aiming at optimizing prevention and treatment of affective dysregulation in children aged 8;0 to 12;11 years. ADOPT consists of five interrelated sub-projects, which will be conducted at several study centers and which use approximately the same assessment instruments. The main objectives of ADOPT include the development and evaluation of new assessment tools as well as investigations concerning the epidemiology of affective dysregulation (including the prevalence of symptoms and comorbid disorders) and psychosocial risk and protective factors (sub-project ADOPT-epidemiology). Moreover, an easy accessible and cost-effective online-treatment program for parents (sub-project ADOPT-online) as well as a modularized treatment program to use in out-patient psychotherapy (sub-project ADOPT-treatment) will be developed and evaluated in randomized-controlled trials. The evaluation of the therapy program will include two different samples of children with affective dysregulation: On the one hand children, who still live in their family homes (ADOPT-treatment), and on the other hand children in out-of-home care (ADOPT-institution).

Organizational Data

- DRKS-ID: DRKS00014581
- Date of Registration in DRKS: 2018/07/04
- Investigator Sponsored/Initiated Trial (IST/IIT): yes
- Ethics Approval/Approval of the Ethics Committee: Approved
- (leading) Ethics Committee No.: 139/18, Ethik-Kommission der Universität Ulm

Health Condition or Problem studied

Free text: Affective Dysregulation

Other: F 34.8 (DSM-V): Disruptive mood dysregulation disorder; NIMH Research Domain Criteria (RDoC) Initiative: Negative emotionality, Frustrative non-reward

Interventions/Observational Groups

- Arm 1: Modular Outpatient Treatment of Affective Dsyregulation and coexisting disorders (PeMOT-AD; 9 moths, 24 sessions in total): The program is leaned on evidence-based cognitive-behavoral therapy (CBT) programs (e.g. Coping Power Program, Cognitive-behavioral Therapy for Anger and Aggression in Children, MATCH-ADTC) and consists of individualized training sessions for children, parents and teachers. In detail, children will be trained in anger control and emotion regulation (module

1), problem solving and social skills (modules 2+3), empathy sensing skills (module 4), organizational skills (module 4) and coping with traumata and negative live events (module 6). In addition, parents and teachers will be instructed to reduce dysfunctional (modules 7+9) and strengthen positive parenting behavior and interactions with the child (modules 8+10).

- Arm 2: Treatment-as-Usual (TAU; control group; 9 months, 24 sessions in total): The treatment will follow accepted guidelines and regular treatment schemes, and will take place in the institutes outpatient clinics or with outpatient practitioners (psychiatrists or psychotherapists).
- Arm 3: Children without affective dysregulation (observation group)

Characteristics

- Study Type: Interventional
- Allocation: Randomized controlled trial
- Blinding: Blinded
- Who is blinded: assessor
- Control: Active control (effective treament of control group)
- Purpose: Treatment
- Assignment: Parallel

Primary Outcome

AD-symptoms: To assess symptoms of affective dysregulation, a newly developed clinician-rated interview (Outcome Measure for Affective Dysregulation: OMAD) to evaluate caregiver (in this sub-project foster parents or child care workers from youth welfare institutions) information about the child will be used. The assessments will take place immediately before starting the treatment (T1) and immediately after closing it (T2), as well as 8 months after that (T3). The rating of the clinician and the assignment to treatment arms will be conducted independently (blinded rating). The interviews will be audiotaped; 10% of them will be evaluated by a second rater (interrater-reliability).

Secondary Outcome

Clinician-rated AD symptoms (children, caregivers, teachers; OMAD), ADHD and conduct disorders (DISYPS-III), other comorbid disorders (CBCL 6-18R; TRF 6-18R), psychological wellbeing oft he child (KIDSCREEN), clinician-rated psychosocial impairment of the child (DISYPS-III). All secondary endpoints will be assessed at T1, T2 and T3.

Countries of Recruitment

DE: Germany

Locations of Recruitment

- University Medical Center: KJP, Ulm
- University Medical Center: KJP, Köln
- University Medical Center: KJP, Dresden
- University Medical Center: KJP, ZI Mannheim
- University Medical Center: KJP, Medizinische Hochschule Brandenburg Neuruppin

Recruitment

- Planned/Actual: Actual
- (Anticipated or Actual) Date of First Enrollment: 2018/10/04
- Target Sample Size: 166
- Monocenter/Multicenter trial: Multicenter trial
- National/International: National

Inclusion Criteria

- Gender: Both, male and female
- Minimum Age: 8 Years
- Maximum Age: 12 Years

Additional Inclusion Criteria

age between 8;0 and 12;11 years; living in out-of-home care; clinician-rated AD symptoms > cut-off (OMAD)

Exclusion Criteria

IQ <80; severe mental disorder (profound developmental disorder, schizophrenia, psychosis, bipolar disorder, obsessive compulsive disorder) causing AD symptoms; insufficient language skills of child and/or caregiver; intensive psychotherapy (ongoing or planned)

Addresses

Primary Sponsor

Universitätsklinik Ulm
Steinhövelstraße 5
89075 Ulm
Germany

Contact for Scientific Queries

Universitätsklinikum Ulm Klinik für Kinder- und Jugendpsychiatrie und -psychotherapie
Mr. Prof. Dr. Michael Kölch
Steinhövelstraße 5
89075 Ulm
Germany
Telefon: 0731 500-61601
Fax: 0731 500-61602
E-Mail: michael.koelch@med.uni-rostock.de
URL der Einrichtung: https://www.uniklinik-ulm.de/kinder-und-jugendpsychiatriepsychotherapie.html

Contact for Public Queries

Universitätsklinik Ulm Klinik für Kinder- und Jugendpsychiatrie und -psychotherapie
Ms. Marion Steiner
Steinhövelstraße 5
89075 Ulm
Germany
Telefon: 0731 500-61768
E-Mail: marion.steiner@uniklinik-ulm.de
URL der Einrichtung: https://www.uniklinik-ulm.de/kinder-und-jugendpsychiatriepsychotherapie.html

Sources of Monetary or Material Support

Public funding institutions financed by tax money/Government funding body (German Research Foundation (DFG), Federal Ministry of Education and Research (BMBF), etc.)
Bundesministerium für Bildung und Forschung Dienstsitz Bonn
Heinemannstr. 2
53175 Bonn
Germany
URL: http://www.bmbf.de

Status

Recruitment Status: Recruiting ongoing
