# Supplementary material for: Affective dysregulation in childhood - optimizing prevention and treatment: protocol of three randomized controlled trials in the ADOPT study
Source: BMC Psychiatry. 2019 Sep 2;19:264. doi: 10.1186/s12888-019-2239-8 (PMC6720991; doi:10.1186/s12888-019-2239-8)
Supplement: Supplementary file 4 — Model written informed consent. (PDF 531 kb) [file 12888_2019_2239_MOESM4_ESM.pdf]

Additional file 4: Model written informed consent

Forschungsprojekt ADOPT Treatment

(„Affektive Dysregulation – Optimierung von Prävention und Therapie; Wirksamkeitsüberprüfung einer individualisierten, modularen Behandlung
von Kindern mit affektiver Dysregulation und komorbiden Störungen)

Hilfen für Kinder mit
Schwierigkeiten bei der Gefühlsregulation

- Informationen über die Studie –

Liebe Eltern, liebe Bezugspersonen,

wir stellen Ihnen im Folgenden den nächsten Schritt unserer Studie vor. Ergänzend zum Informationsgespräch sind hier weitere Informationen zum Ziel und Inhalt der Studie beschrieben. Die vorliegende Einwilligungserklärung erläutert Ihnen den Ablauf und warum wir diese Studie durchführen. Lesen Sie diese Informationen bitte aufmerksam durch. Sie haben die Möglichkeit Fragen zu stellen, wenn Ihnen z. B. etwas unklar ist.

1. Warum führen wir diese Studie durch?

Wie Sie bereits aus dem ersten Studienteil wissen, beschäftigt sich die ADOPT-Studie mit Kindern, die Schwierigkeiten im Umgang mit eigenen negativen Gefühlen haben, was auch als affektive Dysregulation bezeichnet wird.

Wir haben verschiedene Unterstützungsmöglichkeiten für Kinder mit Schwierigkeiten in der Gefühlsregulation entwickelt und möchten diese hinsichtlich ihrer Wirksamkeit überprüfen.

Den ersten Schritt dieses Programms – das Online-Elterntraining – kennen Sie bereits. In einem zweiten Schritt soll nun ein neu entwickeltes Behandlungsprogramm zur intensiven ambulanten Psychotherapie für Kinder mit affektiver Dysregulation bezüglich der Wirksamkeit überprüft werden.

Sponsor der Studie: *Name und Adresse*

Studienleiterin: *Name und Adresse*

2. Wie ist der Ablauf der Studie?

In diesem Teil der Studie soll die Wirksamkeit einer ambulanten psychotherapeutischen Behandlung für Kinder mit Schwierigkeiten bei der Gefühlsregulation untersucht werden. Hierfür wird die psychotherapeutische Behandlung mit Behandlungen verglichen, die für Eltern üblicherweise vor Ort vorhanden sind und von den Eltern selbst gesucht werden („Behandlung wie üblich“). Kinder, bei denen auch nach der Teilnahme der Eltern am Online-Elterntraining Schwierigkeiten in der Gefühlsregulation vorhanden sind, werden per Zufall – vergleichbar mit dem Werfen eines Würfels – einer der beiden Gruppen (ambulante Psychotherapie oder „Behandlung wie üblich“) zugewiesen. Die Wahrscheinlichkeit, an der ambulanten Psychotherapie teilzunehmen, beträgt 50%. Bei der psychotherapeutischen Behandlung handelt es sich um eine Einzeltherapie, die 24 ungefähr wöchentliche Sitzungen umfasst. Die Kinder lernen in dieser Behandlung, wie sie unangenehme Gefühle – auch mit Unterstützung ihrer Eltern – besser steuern können. In die Behandlung werden sowohl die Kinder als auch die Eltern und die Lehrer/innen einbezogen, sofern durch die Familien erwünscht und notwendig. In der psychotherapeutischen Behandlung setzen wir auch – Ihr Einverständnis vorausgesetzt – eine therapieunterstützende App ein. Sie enthält Funktionen, die den Übertrag von

Therapieinhalten in den Alltag erleichtern, aber auch Alltagssituationen für die Therapie leichter zugänglich machen sollen. Zu diesen Funktionen zählen das Führen eines Videotagebuchs, eine Momentaufnahme-Funktion, bei der zu definierten Zeitpunkten die aktuellen Gefühle in Verbindung mit aktuellem Verhalten abgefragt werden, die Erinnerung an individuell vereinbarte Therapieaufgaben, sowie eine Trainingsfunktion der erarbeiteten Inhalte. Für die Benutzung der App wird Ihrem Kind durch uns für den jeweils notwendigen Zeitraum ein Smartphone zur Verfügung gestellt. Dieses ist ausschließlich zur Verwendung der App eingestellt, alle anderen Funktionen sind gesperrt.

Familien in der Gruppe „Behandlung wie üblich“ können je nach Wunsch eigenständig ein Unterstützungsangebot suchen (z. B. bei einer Erziehungsberatungsstelle) oder abwarten, ob sich das Problem von selbst vermindert. Außerdem bieten wir diesen Familien einen zweiten Beratungstermin im Verlauf der Studie (nach ca. 4 Monaten) an. Unabhängig davon, zu welcher Gruppe Sie gehören werden, bitten wir Sie und Ihr Kind, wie bisher Fragebogen auszufüllen und an Interviews teilzunehmen. Dabei geben Sie uns Informationen zu Problemen und Stärken Ihres Kindes sowie zu Ihrem Erziehungsverhalten und Ihrer eigenen Belastung. Die Interviews werden – Ihr Einverständnis vorausgesetzt – auf Video aufgezeichnet.

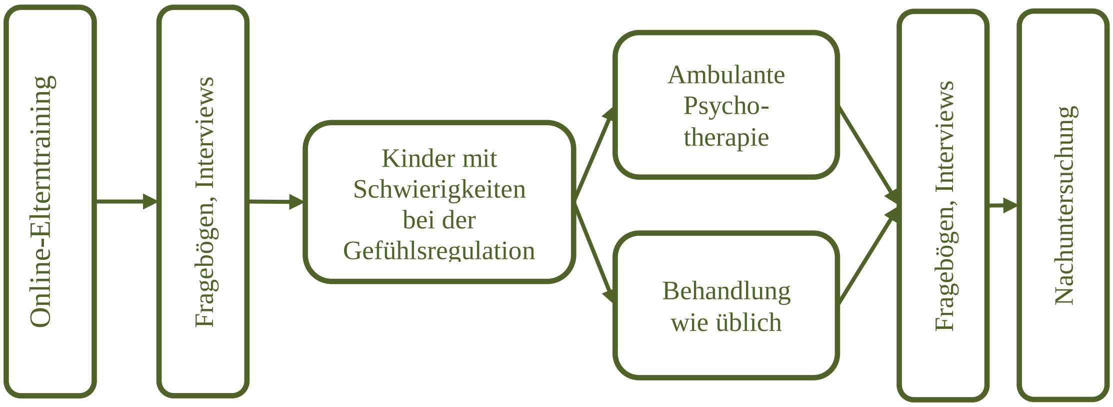

3. Wer ist der Auftraggeber der Studie und wie viele Patient/innen werden an der Studie teilnehmen?

Die Studie wird vom Bundesministerium für Bildung und Forschung (BMBF) gefördert.

Für die Studie wollen wir gemeinsam im Forschungsverbund an fünf Projektstandorten insgesamt ca. 200 Familien gewinnen. An insgesamt fünf Instituten (Uniklinik Köln, Universität Köln, Uniklinik Ulm, Uniklinik Dresden, ZentraIinstitut für seelische Gesundheit Mannheim) sollen die Familien behandelt werden.

4. Wie lange dauert die Teilnahme an der Studie?

Die Teilnahmedauer an diesem zweiten Teil der Studie beträgt in der Regel 6 bis 9 Monate. Während dieser Zeit werden für die Kinder, welche die ambulante Psychotherapie von uns erhalten, insgesamt 24 je 50-minütige Therapiesitzungen stattfinden, an denen entweder die Eltern oder ihr Kind oder beide gemeinsam teilnehmen. Die Sitzungen finden in der Regel einmal wöchentlich statt. Kinder, die eine „Behandlung wie üblich“ erhalten, werden mit ihren Eltern noch einmal während der Studie (ungefähr 4 Monate nach dem ersten Untersuchungstermin) für eine Beratung zu den Schwierigkeiten ins Studienzentrum eingeladen. Um untersuchen zu können, wie lange mögliche Veränderungen bei den Kindern oder in den Familien anhalten, findet die letzte Befragung nach weiteren acht Monaten nach Ende der Behandlung statt.

5. Welches Risiko bzw. welcher Nutzen ist mit der Studienteilnahme verbunden?

Wir können nicht mit Sicherheit sagen, ob Ihr Kind von der Studienteilnahme profitieren wird. Allen durch uns angebotenen Hilfen, die in dieser Studie zum Einsatz kommen, wird jedoch durch Voruntersuchungen bei

ähnlichen Problemen eine gute Wirksamkeit bescheinigt. Die Wirksamkeit des Unterstützungsansatzes bei Kindern mit Schwierigkeiten bei der Gefühlsregulation wurde jedoch bisher nicht genau untersucht. Es ist daher noch unklar, welche Kinder von welchen Hilfen am meisten profitieren. Die gesammelten Informationen aus dieser Studie können uns jedoch helfen, bei anderen Kindern mit diesen Schwierigkeiten künftig noch besser genau die Unterstützung einzuleiten, die im Einzelfall am besten ist.

Die häufige, exzessive und missbräuchliche Nutzung von sogenannten „Smartdevices" (Smartphone, Tablet) kann für Kinder möglicherweise Risiken beinhalten. Während der Teilnahme an unserer Studie wird Ihr Kind, sofern es in der entsprechenden Gruppe behandelt wird, jedoch die Smartphone-App im Rahmen weniger Wochen, täglich nur für einen kurzen Zeitraum nutzen. Alle weiteren Funktionen des Gerätes sind zudem deaktiviert, so dass der Anreiz für eine längere Nutzung sehr gering ist. Für einen vergleichsweise kurzen Nutzungszeitraum sind uns keine Risiken bekannt. Vor der selbständigen Nutzung im Alltag wird mit Ihrem Kind genau besprochen, wie die Therapie-App anzuwenden ist, welche Art von Daten dadurch erfasst werden und welche Folgen sich daraus für den Umgang mit dem Smartphone ergeben. Zum Schutz der Daten vor unautorisierten Personen wird das Handy durch ein Passwort geschützt.

Die Studienleitung wurde von der Ethikkommission *XY* beraten und die Studiendurchführung am *Datum* zustimmend bewertet.

6. Welche anderen Behandlungsmöglichkeiten gibt es außerhalb der Studie

Sie und Ihr Kind müssen nicht an dieser Studie teilnehmen, wenn Sie oder Ihr Kind das nicht wollen. Falls Sie sich gegen eine Studienteilnahme entscheiden, werden wir Sie bei Interesse in einem persönlichen Gespräch zu weiteren Unterstützungsangeboten beraten. Bei Kindern mit Schwierigkeiten bei der Gefühlsregulation kann z. B. eine Beratung der Familie in einer Erziehungsberatungsstelle, eine Anbindung in einer kinder- und jugendpsychiatrischen Ambulanz oder Praxis oder eine Kinder- und Jugendlichenpsychotherapie hilfreich sein.

7. Gibt es eine spezielle Versicherung?

Für die Teilnahme an den vier Messzeitpunkten (inklusive dem, der gerade stattgefunden hat) sind Wegeunfallversicherungen bei der *XY* Versicherung (Adresse, Kontaktdaten) abgeschlossen worden.

<u>Wegeunfallversicherung für den zweiten Termin mit Interview und Fragebogen:</u>

Versicherungszeitraum:	*Datum-Datum*
Versicherung:	*XY Versicherung*
Versicherungsscheinnummer:	*XY*

<u>Wegeunfallversicherung für den dritten bis fünften Termin mit Interview und Fragebogen:</u>

Versicherungszeitraum:	*Datum-Datum*
Versicherung:	*XY Versicherung*
Versicherungsscheinnummer:	*XY*

<u>Inhalt aller Versicherungen:</u>

Ersatzleistung bei Tod:	*Betrag* €
Ersatzleistung bei Invalidität:	*Betrag* €
Bergungskosten (subsidiär):	*Betrag* €
Kosmetische Operationen (subsidiär):	*Betrag* €

Wenn Sie auf dem Weg zum einem Termin oder auf dem Weg vom Termin nach Hause einen Unfall erleiden und die Versicherung in Anspruch nehmen möchten, müssen Sie unverzüglich einen Arzt hinzuziehen, seine Anforderungen befolgen und die o.g. Versicherung unterrichten. Weitere Informationen können Sie den *Allgemeinen Bedingungen für die SV Unfallversicherung* entnehmen, die Sie von uns erhalten haben. Bei der Anzeige des Unfalls gegenüber der Versicherung können die Projektmitarbeiter/innen Sie unterstützen. Wenn Sie Ihre Anzeige direkt an den Versicherer richten, informieren Sie bitte zusätzlich die Projektmitarbeiter/innen. Bei der Aufklärung der Ursache oder des Umfangs eines Schadens sind Sie zum Mitwirken verpflichtet.

Da es keine weiteren Risiken durch diese Hilfe gibt, sind keine weiteren Versicherungen nötig.

Für die Therapie-Smartphones wird eine separate Geräteversicherung abgeschlossen.

8. Entstehen für mich Kosten durch die Teilnahme an der Studie?

Die Kosten für die Anfahrt zu den Untersuchungsterminen werden durch die Studienteilnehmer selbst getragen.

Die Behandlungskosten werden von der zuständigen Krankenkasse übernommen, weil Ihr Kind behandlungsbedürftige Verhaltensprobleme zeigt.

9. Ist die Studienteilnahme freiwillig?

Die Teilnahme Ihres Kindes und von Ihnen an dieser Studie ist freiwillig. Wenn Sie sich zur Teilnahme entscheiden und die Einwilligungserklärung unterschreiben, können Sie und Ihr Kind die Studie jederzeit, ohne Angabe von Gründen abbrechen.

Selbst wenn Sie Ihr Einverständnis geben, wird die Entscheidung Ihres Kindes respektiert, nicht an der Studie teilzunehmen bzw. die Teilnahme an der Studie vorzeitig zu beenden.

Die Studienmitarbeiter/innen werden Sie über alle neuen Erkenntnisse, Änderungen und wichtigen Informationen, die in Bezug auf diese Studie bekannt werden und die für Ihr Kind von wesentlicher Bedeutung sein könnten, umgehend informieren. Auf dieser Basis können Sie dann die Entscheidung zur weiteren Teilnahme an dieser Studie neu überdenken.

10. Kann die Teilnahme an der Studie auch durch Andere vorzeitig beendet werden?

Es ist möglich, dass der/die Therapeut/in entscheidet, die Teilnahme Ihres Kindes an der Studie vorzeitig zu beenden, ohne vorher Ihr Einverständnis einzuholen. Gründe hierfür können sein, dass:

- die Probleme Ihres Kindes sich so stark verschlechtern, dass eine andere Behandlungsform notwendig erscheint.
- den Erfordernissen der Studie nicht ausreichend nachgekommen wird/werden kann.
- neue Informationen darauf hinweisen, dass die Behandlung für Ihr Kind nicht länger sinnvoll ist.

Ihr/e Therapeut/in wird dann die Situation und die weitere Vorgehensweise mit Ihnen besprechen.

11. Was geschieht mit Ihren Daten?

Während der Studie werden Informationen von Ihnen erhoben, niedergeschrieben und elektronisch gespeichert. Hierbei handelt es sich sowohl um Ihre Daten als auch um die Daten Ihres Kindes. Die für die Studie wichtigen Daten werden zusätzlich in pseudonymisierter Form gespeichert, ausgewertet und gegebenenfalls weitergegeben. Pseudonymisiert bedeutet, dass keine Angaben von Namen oder Initialen verwendet werden, sondern nur ein Nummern- und/oder Buchstabencode. Videodateien, die während der Nutzung der Therapie-App aufgezeichnet werden, sind nicht im gleichen Maße unkenntlich zu machen. Diese sind nur von Studienmitarbeiter/innen einsehbar und werden separat und verschlüsselt gespeichert.

Die Daten sind gegen unbefugten Zugriff gesichert. Eine Entschlüsselung erfolgt nur unter den vom Gesetz vorgeschriebenen Voraussetzungen. Die Speicherung der erhobenen Daten erfolgt in Verantwortung von *Name, Adresse*.

Sie haben das Recht, Einblick in Ihre Daten zu nehmen, die während der Studie erhoben werden. Sollten Sie dabei Fehler in Ihren Daten feststellen, so haben Sie das Recht, diese durch Studienmitarbeiter/innen korrigieren oder löschen zu lassen.

Die Daten, die mit der App erhoben werden, können jederzeit eigenmächtig gelöscht werden.

Weitere Einzelheiten, insbesondere zur Möglichkeit eines Widerrufs, entnehmen Sie bitte der Einwilligungserklärung im Anschluss an diese Studieninformation.

12. An wen wende ich mich bei weiteren Fragen?

Für weitere Fragen im Zusammenhang mit dieser Studie stehen Ihnen die Studienmitarbeiter/innen gerne zur Verfügung. Auch Fragen, welche die Rechte Ihres Kindes als Teilnehmer/in an dieser Studie oder die Aufbewahrung bzw. Vernichtung von persönlichen Daten betreffen, werden wir Ihnen gerne beantworten. Wenn Sie, Ihr Kind oder Ihre Angehörigen Fragen oder Bedenken zu dieser Studie, zu möglichen Risiken und unerwünschten Nebenwirkungen der eingesetzten Formen der Hilfe oder zu Ihren Rechten haben oder wenn Sie zusätzliche Informationen wünschen oder studienbezogene Schädigungen melden möchten, können Sie und Ihr Kind jederzeit die Studienmitarbeiter/innen kontaktieren.

**Kontakt Studienleitung:**	**Kontakt vor Ort**
Kontaktdaten	*Kontaktdaten*

Ihr Team von ADOPT Treatment

Forschungsprojekt ADOPT Treatment

Hilfen für Kinder mit
Schwierigkeiten bei der Gefühlsregulation

Einwilligungserklärung für Sorgeberechtigte

Sie haben schriftlich Informationen zu der ADOPT-Studie von uns erhalten und wir haben mit Ihnen persönlich und ausführlich über den Studienablauf gesprochen. Mit Ihrer Unterschrift bestätigen Sie uns die folgenden Aussagen:

- Ich habe die Probandeninformation erhalten und gelesen und Ziel, Ablauf und Durchführung der Studie verstanden. Ich wurde über Wesen, Bedeutung, Tragweite und Risiken der geplanten Studienteilnahme informiert. Mir wurde ausreichend Gelegenheit gegeben, alle offenen Fragen zu klären. Ich habe jederzeit das Recht, weitere Informationen zur Studie zu erhalten.
- Ich bin bereit, an der Studie teilzunehmen und zu mehreren Zeitpunkten Fragebogen auszufüllen und an zwei Interviewterminen teilzunehmen. Mein Kind füllt ebenfalls Fragebogen aus und nimmt an Interviews teil.
- Ich bin damit einverstanden, dass mein Kind über eine Zufallszuweisung (Randomisierung) einer Interventionsgruppe (ambulante Psychotherapie oder Behandlung wie üblich) zugeordnet wird.
- Wenn mein Kind in der Gruppe „ambulante Psychotherapie" ein für die Studie aufgesetztes Test-Smartphone nutzt, werde ich es daran erinnern, dieses pfleglich zu behandeln.
- Wenn mein Kind in der Gruppe „ambulante Psychotherapie" ein für die Studie aufgesetztes Test-Smartphone nutzt, weise ich mein Kind daraufhin, dass es bei den Aufnahmen für das Videotagebuch nur sich selbst und keine weiteren Personen filmt.
- Ich und/oder mein Kind haben jederzeit das Recht, ohne Angabe von Gründen von der Teilnahme an der Studie zurückzutreten, ohne dass dadurch Nachteile für mich oder mein Kind entstehen. In diesem Fall werden die bis zu diesem Zeitpunkt erhobenen Daten gelöscht oder vollständig anonymisiert, es sei denn, ich stimme/wir stimmen nach erfolgtem Widerruf der weiteren Verwendung der bislang erhobenen Daten ausdrücklich zu.
- Ich wurde darüber informiert, dass die Interviews im Rahmen der Messzeitpunkte auf Video aufgezeichnet werden.
- Zu Verarbeitung und zum Schutz unserer Daten wurden mir in der hier nachfolgend abgedruckten Datenschutzerklärung stehenden Zusagen gemacht.

Datenschutz:

Mir/Uns ist bekannt, dass bei dieser Studie personenbezogene Daten über mich/uns und mein/unser Kind erhoben, gespeichert und ausgewertet werden sollen. Dabei handelt es sich insbesondere über medizinische Befunde über das Kind.

Die Verwendung der Angaben über die Gesundheit des Kindes erfolgt nach gesetzlichen Bestimmungen und setzt vor der Teilnahme an der Studie folgende freiwillig abgegebene Einwilligungserklärung voraus, das heißt, ohne die nachfolgende Einwilligung kann das Kind nicht an der Studie teilnehmen.

Unsere persönlichen Daten werden in Übereinstimmung mit dem geltenden Datenschutzgesetz vertraulich behandelt. Sie werden in pseudonymisierter Form gespeichert. Das bedeutet, dass Identifikationsmerkmale (z. B. Name, Anschrift) durch eine Codenummer ersetzt werden, sodass eine Zuordnung der Fragebogendaten zu einer bestimmten Person nur über weitere Hilfsmittel (Referenzliste) möglich ist. Diese Verbindung wird lediglich für die Koordination der Erhebung benötigt und anschließend vernichtet. Die wissenschaftliche Auswertung der Daten durch den Sponsor (*Name*) oder eine von diesem beauftragte Stelle erfolgt so, dass kein Rückschluss auf mich oder mein Kind möglich ist. Ein Rückschluss ist nur dann theoretisch möglich, wenn die Person, die die Daten auswertet, auch über die Referenzliste zum Entschlüsseln der Pseudonymisierung verfügt. Mir ist bewusst, dass die Aufnahmen, die mein Kind im Rahmen der App-Nutzung bei der Aufzeichnung des Video-Tagebuchs tätigt, nicht in der gleichen Form wie die übrigen Daten unkenntlich gemacht werden können. Diese Daten werden jedoch nur von autorisierten Mitarbeitern der Studie zur wissenschaftlichen Auswertung eingesehen und unter Einhaltung besonderer Schutzmaßnahmen verschlüsselt separat gespeichert. Von den Ergebnissen der Auswertung kann kein Rückschluss mehr auf mein Kind oder mich gezogen werden. Im Falle der Veröffentlichung von Studienergebnissen bleibt die Vertraulichkeit unserer persönlichen Daten damit gewährleistet.

Die im Rahmen dieser Studie erhobenen Daten, insbesondere Angaben über die Gesundheit des Kindes, werden von einem elektronischen Datensystem erfasst und statistisch ausgewertet. Nach Ende der Studie werden alle Daten nach den derzeit gültigen Richtlinien entsprechend gespeichert und archiviert.

Ich habe das Recht, Einblick in die zu mir und meinem Kind im Rahmen der Studie erfassten Daten zu nehmen sowie eine unentgeltliche Kopie zu erhalten. Sollte ich dabei Fehler feststellen, habe ich das Recht, diese korrigieren zu lassen. Unsere Daten werden über einen Zeitraum von mindestens 10 Jahren in einem sicheren System gespeichert und im Anschluss gelöscht. Die Beachtung des Bundesdatenschutzgesetzes ist in vollem Umfang sichergestellt.

Ich erkläre mich damit einverstanden, dass Personen, die die Aufgabe haben, den Projektablauf zu kontrollieren, Zugang zu den personenbezogenen Daten meines Kindes erhalten, wenn dies für die ordnungsgemäße Durchführung notwendig ist.

Ich habe das Recht auf Widerruf hinsichtlich der Datenverarbeitung. In diesem Fall werden die bis zu diesem Zeitpunkt erhobenen Daten gelöscht oder vollständig anonymisiert, es sei denn, ich stimme/wir stimmen nach erfolgtem Widerruf der weiteren Verwendung der bislang erhobenen Daten ausdrücklich zu. Ich bin/wir sind bereits darüber aufgeklärt worden, dass das Kind die Teilnahme an der Studie jederzeit beenden kann.

Außerdem erkläre ich mich damit einverstanden, dass autorisierte und zur Verschwiegenheit verpflichtete Beauftragte des Sponsors zum Zwecke der Qualitätssicherung sowie Beauftragte der Ethikkommission zum Zwecke des Monitorings in die beim Studienleiter vorhandenen personenbezogenen Daten meines Kindes Einsicht nehmen, soweit dies für die Überprüfung der ordnungsgemäßen Durchführung der Studie notwendig ist. Für diese Maßnahme entbinde ich die im Rahmen der Studie für mich bzw. mein Kind zuständigen Studienmitarbeiter von der ärztlichen Schweigepflicht.

Die Speicherung und Auswertung der erhobenen Daten erfolgt in Verantwortung von *Name* (*Adresse*).

Der zuständige Datenschutzbeauftrage für das leitende Prüfzentrum ist *Name*, *Adresse*. Der zuständige Datenschutzbeauftrage für *Prüfzentrum* ist *Name*, *Adresse*.

Mir ist bekannt, dass ich berechtigt bin, mich bei einer Datenschutz-Aufsichtsbehörde zu beschweren. Ich kann mich in diesem Fall bei der Landesbeauftragen für Datenschutz und Informationsfreiheit Bundesland (*Name*, *Adresse*) oder bei *Datenschutzaufsichtsbehörde des Prüfzentrums mit Kontaktdaten* beschweren.

Haben Sie noch Fragen?

Wenden Sie sich an uns - wir beantworten alle Ihre Fragen sehr gern!

Homepage: www.adopt-studie.de

– Einwilligungserklärung für Sorgeberechtigte (ADOPT Treatment) –

Ich habe die obigen Informationen gelesen.

- Ich habe verstanden, warum die Studie gemacht wird und auch, was gemacht wird. Ich kann jederzeit weitere Informationen zur Studie bekommen.
- Ich bin damit einverstanden, dass mein Kind über Zufallsverteilung (Randomisierung) einer Interventionsgruppe (ambulante Psychotherapie oder Behandlung wie üblich) zugeordnet wird.
- Ich möchte mit meinem Kind an der Studie teilnehmen und weiß, dass dies freiwillig ist. Ich und/oder mein Kind können auch später zu jeder Zeit sagen, wenn ich/wir nicht mehr teilnehmen möchte/n. Mir oder meinem Kind werden dadurch keine Nachteile entstehen.
- Ich bin damit einverstanden, dass mein Kind an der Studie teilnimmt.
- Meine Daten und die Daten meines Kindes werden verschlüsselt unter einem Code gespeichert und untersucht.
- Mir wurden die Versicherungsbestätigungen und -bedingungen der Wegeunfallversicherungen schriftlich ausgehändigt.
- Ich bin damit einverstanden, dass die Interviews im Rahmen der Messzeitpunkte auf Video aufgezeichnet werden (bitte zutreffendes ankreuzen).

  ☐ nein ☐ ja Ich habe verstanden, dass es nicht möglich ist, Videodaten so zu anonymisieren, dass nicht mehr erkennbar ist, welche Person auf dem Video zu sehen ist. Ich weiß, dass die Videodateien nur von autorisierten Mitarbeitern der Studie gesehen werden dürfen.

- Wenn mein Kind der Gruppe „ambulante Psychotherapie“ zugeordnet wird, bin ich damit einverstanden, dass mein Kind für einen gewissen Zeitraum ein Test-Smartphone nutzt, welches für die Studie präpariert wurde. Darüber hinaus bin ich dazu bereit einen separaten Leihvertrag zu unterzeichnen. Ich wurde darauf hingewiesen, dass bei den Aufzeichnungen der Video-Tagebuchfunktion in der Smartphone-App nur mein Kind und keine weiteren Personen gefilmt werden sollen. Auch mit meinem Kind wurde dies besprochen.

  ☐ nein ☐ ja Ich habe verstanden, dass es nicht möglich ist, Videodaten so zu anonymisieren, dass nicht mehr erkennbar ist, welche Person auf dem Video zu sehen ist. Ich weiß, dass die Videodateien nur von autorisierten Mitarbeitern der Studie gesehen werden dürfen.

____

Name des Kindes in Druckbuchstaben

☐ Ich entbinde/Wir entbinden das Studienpersonal von der Schweigepflicht gegenüber dem/r Lehrer/in _________________________ (Name der Lehrkraft, die in die Behandlung einbezogen werden soll) und erkläre mich/erklären uns damit einverstanden, dass der/die Lehrer/in gegenüber dem Studienpersonal Informationen bezüglich des Verhaltens des Kindes weitergeben darf.

Für die Teilnahme des Kindes an der Studie ist die Zustimmung beider Sorgeberechtigter erforderlich. Falls Sie das alleinige Sorgerecht besitzen, kreuzen Sie bitte hier an:

☐ Ja, ich habe die Alleinsorge für das genannte Kind.

________________________________ ________________________________

Ort, Datum Name & Unterschrift **erste/r Sorgeberechtigte/r**

________________________________ ________________________________

Ort, Datum Name & Unterschrift **zweite/r Sorgeberechtigte/r**

(ggf. durchstreichen)

Ich habe das Aufklärungsgespräch geführt und die Einwilligung der Erziehungsberechtigten eingeholt.

____

Name des/r Studienmitarbeiter/in in Druckbuchstaben

Prüfzentrum: ____

____

Datum Unterschrift des/der aufklärenden Studienmitarbeiters/in
